# *Aframomum melegueta* Seed Extract’s Effects on Anxiety, Stress, Mood, and Sleep: A Randomized, Double-Blind, Pilot Clinical Trial

**DOI:** 10.3390/ph18020278

**Published:** 2025-02-19

**Authors:** Rubén Pérez-Machín, Tanausú Vega-Morales, Carlos Elvira-Aranda, Loreto Lledó-Rico, María José Gomis-Gomis, Laura López-Ríos

**Affiliations:** 1 Nektium Pharma S.L., C/Las Mimosas 8, Polígono Industrial Arinaga, 35118 Las Palmas, Spain; tvega@nektium.com (T.V.-M.); llopez@nektium.com (L.L.-R.); 2 Kinetic Performance S.L., Scientific Park of Alicante, 03690 Alicante, Spain; carlos.elvira@kineticperformance.es (C.E.-A.); loret1996@gmail.com (L.L.-R.); mjgomisg@gmail.com (M.J.G.-G.); 3 Research Group on Physical Activity Sciences and Sport, Education School, University of Alicante, 03690 Alicante, Spain

**Keywords:** *Aframomum melegueta*, botanical extract, endocannabinoid system, phytocannabinomimetics, stress, anxiety, vanilloid compound, paradol

## Abstract

**Background and aims:** *Aframomum melegueta* (*A. melegueta*) from the ginger family is appreciated for its pungent seeds widely used in African ethno-medicine. Among the several biological activities associated with the seed’s preparations, some preclinical studies suggest a set of neuroactive properties that have not been tested in humans to date. We performed a clinical trial to investigate the effects of *A. melegueta* seed extracts on anxiety, stress, mood, and sleep in healthy subjects with moderate anxiety levels. In vitro pharmacological assays targeting the endocannabinoid, serotoninergic, and GABAergic systems were conducted to elucidate the underlying mechanism of action. **Methods:** *A. melegueta* standardized to 10% total vanilloids (primarily 6-gingerol, 6-shogaol, and 6-paradol) was obtained after hydroalcoholic extraction and the spray-drying microencapsulation process. Subjects consumed 50, 100, or 150 mg of the extract daily for two days. A set of validated psychometric test questionnaires was collected before and 48 h after the first intake. *A. melegueta* extract interaction with canonical endocannabinoid receptors (hCB1R and hCB2R), the serotonin receptor (5HT1AR) and gamma-aminobutyric acid receptor (GABAA1R) was evaluated by the radioligand binding assay. Additionally, receptor functional assays and enzyme inhibition assays were conducted to test the extract’s functional activity on the non-canonical endocannabinoid receptor (TRPV1) and the cannabinoid fatty-acid amide hydrolase enzyme (FAAH), respectively. **Results:** In vitro pharmacological tests showed that the *A. melegueta* extract activated TRPV1, modulated both hCB2R and 5HT1AR and inhibited FAAH, which is the enzyme primarily responsible for hydrolyzing endogenous anandamide. After a 48 h intake period, the extract significantly reduced anxiety and tension related to stress, improved overall mood, and enhanced sleep quality in the participants at doses ranging from 50 to 150 mg, with no reported side effects. **Conclusions:** This study supports the potential of the *A. melegueta* extract for anxiety reduction, mood improvement, stress mitigation, and sleep enhancement. The in vitro tests suggest that the extract’s primary mechanism of action may involve the inhibition of FAAH, which is a key target in anxiety management.

## 1. Introduction

Stress is an inherent aspect of human life, encompassing a range of physiological and psychological reactions that occur when an individual perceives a stimulus as threatening or challenging. Fortunately, humans have developed the capacity to adapt to various demanding or unpredictable stressors (whether physical, psychosocial, or environmental) through the activation of intricate physiological mechanisms collectively referred to as the “stress response”. The neural stress system detects events and interprets stressors as either actual or potential threats, triggering the rapid activation of the sympathetic adrenomedullary (SAM) axis and the hypothalamus–pituitary–adrenal (HPA) axes—the two major systems responsible for orchestrating the stress response. This activation results in the initiation of physiological and behavioral mechanisms that maintain or restore homeostasis and promote adaptation to stressors [1,2].

While there is a consensus that moderate stress levels (eustress) are beneficial, prolonged or repeated exposure can overwhelm the stress response system, leading to an inability to achieve homeostatic balance (distress). This stress overload can manifest in a range of symptoms, including cardiometabolic, gastrointestinal, and psychological disturbances, such as nervousness, depression, anxiety, and insomnia [3]. According to the World Health Organization (WHO), “Stress makes it hard for us to relax and can be accompanied by a range of emotions, including anxiety and irritability” and chronic stress, can lead to anxiety and depression [4]. Current strategies for managing stress-related symptoms include non-pharmacological interventions (e.g., cognitive–behavioral approaches), pharmacological treatments (e.g., antidepressants, anxiolytics, and beta-blockers), and the use of nutraceutical botanical extracts. An ideal approach would address multiple symptoms holistically while maintaining a favorable safety profile. Prescription antidepressants and anxiolytics are typically reserved for more severe conditions, such as major depressive disorder or generalized anxiety disorder, and come with a serious side-effect profile and the potential for dependency. Selected botanical preparations may serve as suitable alternatives to pharmaceuticals for cases of transient, mild-to-moderate stress, helping to alleviate physical symptoms, such as fatigue (by increasing or maintaining energy reserves), and psychological symptoms, such as low mood, anxiety, and sleep disturbances. Common dietary botanicals are generally well tolerated and pose a minimal risk of dependence.

Among the diverse range of botanicals from the African continent, the seeds of *A. melegueta*, a member of the *Zingiberaceae* family [5]—commonly known as grains of paradise, alligator pepper, or melegueta pepper—are traditionally used in West Africa as a pungent spice in cooking [6] as well as in traditional African medicine for the management of a wide range of conditions [7,8]. The seeds are rich in pungent vanilloid-type compounds (1.5–3%), primarily 6-gingerol, 6-shogaol, and 6-paradol, along with smaller amounts of flavonoids, diarylheptanoids, essential oils, and alkaloids [9,10,11,12].

Over the past three decades, the biological activities of *A. melegueta* seeds and related extracts have been investigated through in vitro studies, preclinical research, and some human clinical trials [13]. Numerous studies have attempted to elucidate the phytochemical profile of *A. melegueta* seeds to link them to their functional properties. For example, Ojo et al. (2018) demonstrated the ability of specific *A. melegueta* seed extracts to act as neuromodulators in rodent models, reporting antidepressant and neuroprotective effects from an ethanolic extract of *A. melegueta* in the forced swim test [14]. In contrast, Umukoro and Ashorobi (2006) reported negative results in a similar forced swim endurance test in mice, which evaluated the anti-stress potential of an aqueous extract of *A. melegueta* seeds [15]. This suggests that the more lipophilic phytochemical fraction may be crucial for the anti-stress and anxiolytic activity associated with *A. melegueta* preparations.

Additionally, recent evidence of the effects of *A. melegueta* seeds on the CNS was provided by López-Ríos and co-workers (2021), who reported electropharmacogram (EEG) profiles for various doses of ethanolic *A. melegueta* seed extracts in conscious, freely moving rats [16]. The electropharmacogram profile of the *A. melegueta* seed extract was compared with the profiles of reference pharmaceuticals using discriminant analysis. The authors observed a close match between the profile of the *A. melegueta* seed extract and that of rolipram (a selective phosphodiesterase IV inhibitor with anxiolytic, antidepressant, and neuroprotective activity), providing preliminary in vivo evidence of the extract’s anxiolytic and antidepressant potential.

Moreover, the inhibitory activity of alkaloid-rich extracts obtained from *A. melegueta* seeds on enzymes such as acetylcholinesterase (AChE), the angiotensin-1-converting enzyme (ACE), phosphodiesterase-5 (PDE-5), and arginase has been evaluated, suggesting the potential for enhancing memory and cognition [12]. Additionally, Okeke et al., (2018) suggested that these extracts could aid in the development of new erectogenic agents [17].

Given the CNS modulatory activity of *A. melegueta* seeds and their extracts [12,14], the present clinical study aimed to evaluate the efficacy of a proprietary microencapsulated ethanolic extract of *A. melegueta*, known as Vanizem^®^ (VAN), in reducing anxiety, relieving stress, improving mood, and enhancing sleep quality in middle-aged individuals. This study also sought to determine the effective dose. Additionally, in vitro studies on selected CNS targets were conducted to gain further insight into its mechanisms of action, focusing on key targets within the serotoninergic, GABAergic, and endocannabinoid systems.

## 2. Results

### 2.1. A. melegueta Seed Extract Evaluation Using In Vitro Pharmacology Assays

**Figure 1:**
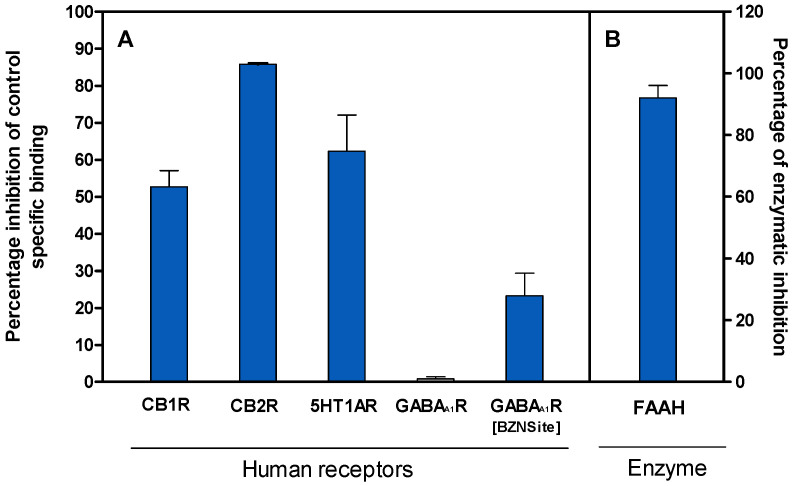
(**A**) The radioligand binding assay of VAN against several receptors in the nervous system. A tritiated specific ligand for each receptor was incubated with VAN at a concentration of 200.3 μg/mL. The competitive displacement of the tritiated ligands by VAN was measured via the scintillation counting of the bound radio-isotopic ligand to its specific receptor. VAN binding was calculated as a percentage of the inhibition of the radioligand specific to each receptor. (**B**) The inhibitory activity of VAN on the recombinant FAAH, assessed using a fluorescence-based assay. The percentage of inhibition was determined at 200.3 μg/mL of VAN relative to the vehicle control. Data are expressed as the mean ± SD of three replicates.

To assess the in vitro binding and inhibitory activity of VAN on molecular targets within the eCBs, the extract was screened in a binding assay at a concentration of 200.3 μg/mL (20.03 μg/mL of total vanilloids). As shown in Figure 1, VAN exhibited 92 and 85.5% significant inhibition against FAAH and hCB2R, respectively.

In addition, VAN presented a moderate inhibition of 62.30% against 5HTR1A and 56.6% for human cannabinoid receptor type 1 (hCB1R). In contrast, no significant VAN interaction for GABA binding sites on GABAA1R was detected, as observed with the inhibition percentages for GABAAR (1%) and the GABAAR–benzodiazepine binding site (GABAAR-BZN) (23%) after incubation with the botanical extract.

**Figure 2:**
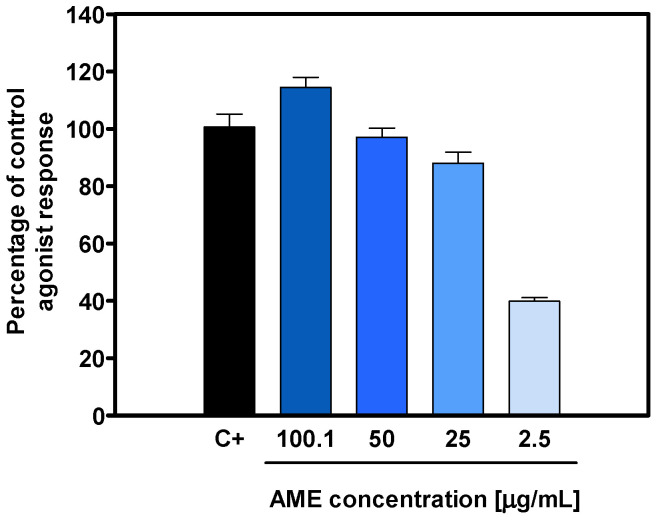
The dose–response of VAN upon TRPV1 activation was assessed using an in vitro agonist functional bioassay. The activity of the control agonist, capsaicin (1 μM), was included as a positive control and set at 100%.

TRPV1 is an ion channel activated by capsaicin, the most well-known vanilloid. Using an increasing concentration of VAN, a dose-dependent activation effect ranging from 41 to 120% of the extract was observed against this ion-channel-associated receptor, indicating that the primary components responsible for this activation are the vanilloid-type phytochemicals present in the botanical extract (Figure 2). The TRPV1 functional agonist capsaicin (1 μM) was used as a positive control, with the results standardized to 100%.

### 2.2. Effect of A. melegueta Seed Extract in Human Clinical Trial

#### Trial Design and Baseline Characteristics

**Figure 3:**
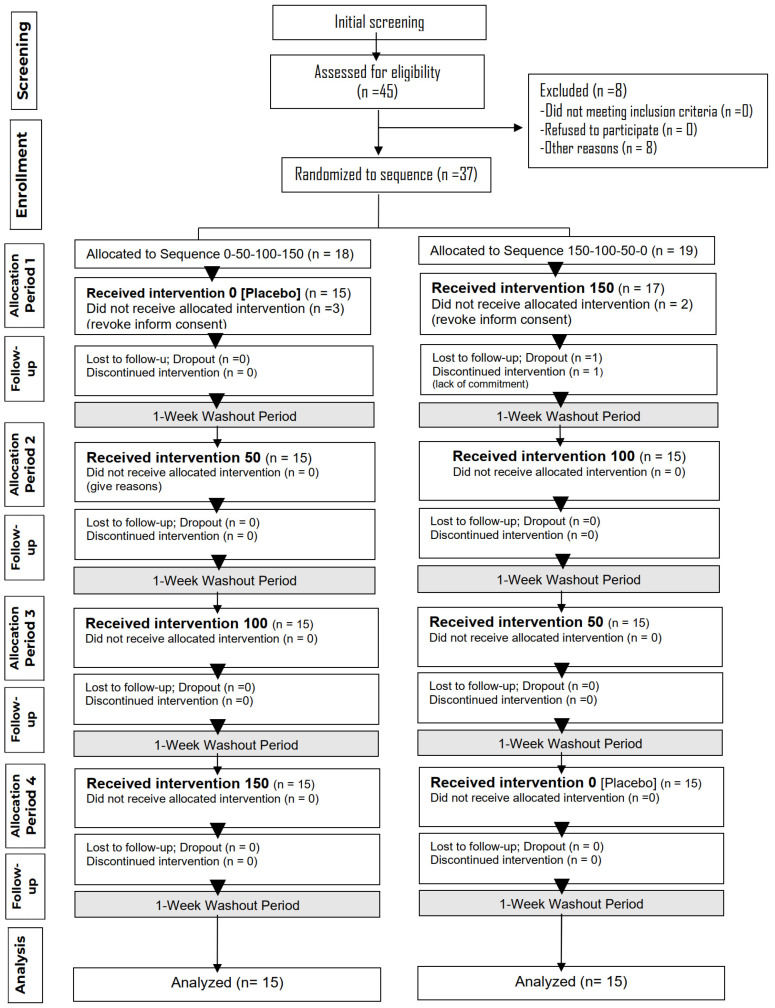
CONSORT flow diagram showing the progression of participants through each stage of the randomized crossover trial.

In total, 45 adults aged 40 to 50 years were recruited at baseline, with 37 participants randomized (18 subjects in Group A and 19 in Group B). Of these, 30 participants completed the study (15 subjects in Group A and 15 in Group B), 44% of whom were women. The CONSORT flow diagram for the crossover design [18] is described in Figure 3. Participants who withdrew from the study either voluntarily chose to discontinue or were lost to follow-up. In addition, The CONSORT document composed of a 25-item checklist, which focuses on the reporting of the trial design, analysis, and interpretation can be consulted in Supplementary Materials (see Consort Check list, File S1).

The demographic parameters of the subjects at baseline are presented in Table 1. The mean age of participants (±SE) was 46.37 ± 2.70 years, with no significant differences between genders.

### 2.3. Efficacy Results

VAN demonstrated a significant improvement across all three study outcomes compared with the placebo group: anxiety scores (m-HAM-A), emotional state (POMS), and sleep quality (LSEQ). The effect size was dose-dependent when mean scores from days 1 to 3 were compared. VAN was well tolerated by the study population, with no adverse effects observed.

#### 2.3.1. Anxiety Score

**Figure 4:**
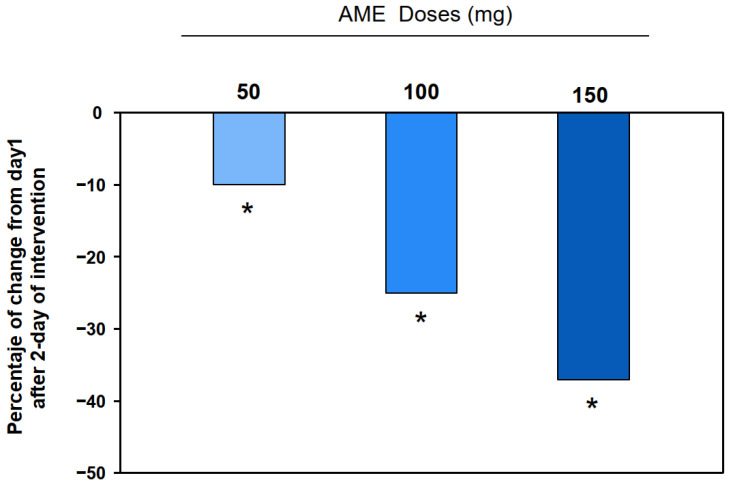
The percentage change (decrease) in anxiety scores (mHAM-A) for the VAN-treated groups compared to the placebo after 2 days of intervention (day 3) (*n* = 30). Significant differences between the placebo and the 50, 100, and 150 mg VAN-treated groups (* *p* < 0.05) were determined at 48 h after intervention (day 3) using ANOVA.

Anxiety scores measured via the m-HAM-A were significantly and progressively reduced with VAN doses ranging from 50 to 150 mg after 3 days of treatment, whereas no significant changes were observed in the placebo group. Notably, participants receiving the 100 mg dose reduced their m-HAM-A score below the threshold for anxiety by the end of the pilot study (Table 2). The percentage reduction in anxiety scores increased with higher doses. After three days of treatment, a 10.27% reduction in anxiety was observed with the 50 mg dose (*p* < 0.05), a 25.01% reduction with the 100 mg dose (*p* < 0.01), and a 37.09% reduction with the 150 mg dose (*p* < 0.01) (Figure 4).

#### 2.3.2. Emotional State

The Profile of Mood States (POMS) results are shown in Table 3. Significant reductions in total mood disturbance (TMD) and tension scores were observed across all groups, including the placebo group (Table 3). Notably, significant reductions in fatigue scores were observed with VAN doses of 50 mg (2.13 ± 1.36 vs. 1.97 ± 1.35; *p* < 0.05), 100 mg (2.00 ± 1.34 vs. 1.70 ± 1.35; *p* < 0.05), and 150 mg (2.03 ± 1.30 vs. 1.50 ± 1.20; *p* < 0.01). Significant reductions in depression scores were also observed with VAN doses of 50 mg (8.27 ± 3.84 vs. 7.07 ± 3.61; *p* < 0.01), 100 mg (7.73 ± 3.50 vs. 5.90 ± 2.89; *p* < 0.01), and 150 mg (8.17 ± 3.47 vs. 5.33 ± 2.89; *p* < 0.01).

For the vigor score, which reflects a positive emotional state, significant increases relative to the placebo were noted for the VAN 100 mg dose (10.37 ± 1.81 vs. 11.07 ± 1.41; *p* < 0.01) and the VAN 150 mg dose (10.37 ± 1.69 vs. 11.27 ± 1.17; *p* < 0.01).

**Figure 5:**
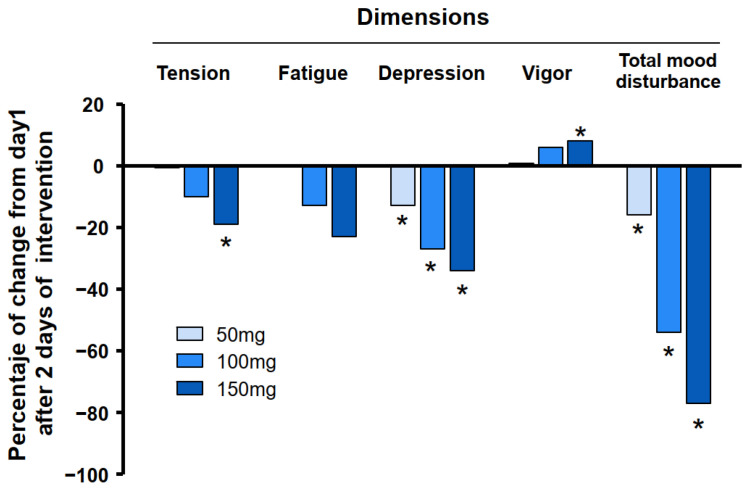
The percentage change in VAN doses compared to the placebo after intervention (day 3) for POMS dimensions. Significant differences between the placebo and 50, 100, and 150 mg of VAN doses (* *p* < 0.05) were determined at 48 h after intervention using ANOVA.

When comparing the different VAN doses and each dose with the placebo after three days of intake, the percentage of improvement (reduction in feelings of discomfort) was significant for all VAN doses in terms of TMD, representing overall mood (16.51, 54.20, and 77.58% for VAN doses of 50, 100, and 150 mg (*p* < 0.01 for all)) (Figure 5). For all dimensions, the percentage of improvement increased with higher VAN doses, with the 150 mg dose showing a significant improvement for tension (19.82%; *p* < 0.05), depression (34.76%; *p* < 0.01), and vigor (8.05%; *p* < 0.05). Significant improvements were also observed for depression with VAN doses of 100 mg (27.78%; *p* < 0.01) and 50 mg (13.46%; *p* < 0.05) (Figure 5).

Significant differences were observed between the 100 and 150 mg VAN doses compared to the 50 mg dose for tension, depression, vigor, and TMD (ANOVA, *p* < 0.05). However, for general mood, a significant difference was found between the 100 and 150 mg VAN doses (*p* < 0.01).

#### 2.3.3. Sleep Evaluation Questionnaire (LSEQ)

Sleep quality was assessed using the LSEQ. No significant changes were observed in the score for “Getting to Sleep” (GTS), “Quality of Sleep” (QOS), and “Alertness and Behavior Following Wakefulness” (BFW) in the placebo group (Table 4). After three days of treatment, there were significant differences in all dimensions for the 100 and 150 mg VAN doses.

**Figure 6:**
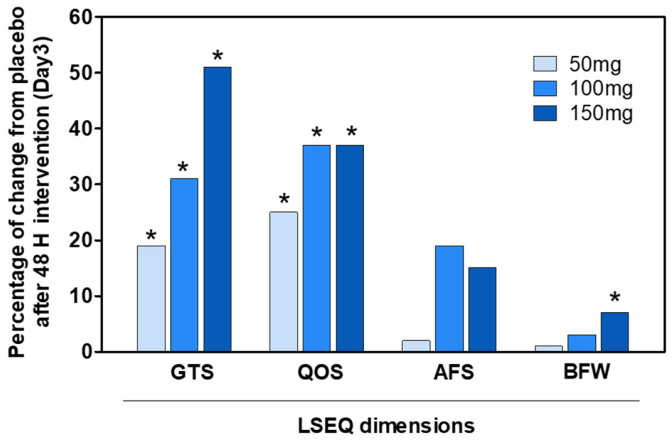
The percentage change in VAN doses compared to the placebo after 48 h of intervention (day 3) for LSEQ dimensions. Significant differences between the placebo and 50, 100, or 150 mg of VAN doses (* *p* < 0.05) were determined at 48 h after intervention using ANOVA.

The results in Figure 6, shown as the percentage of improvement compared to the placebo after three days of treatment, reflect the daily assessment of dimensions with significant differences. The percentage of improvement over the placebo after three days was significant for all doses of GTS (Figure 6). The 50 mg VAN dose, in the short term, improved the ease of falling asleep by 19.41% compared to the placebo and improved sleep quality by 25.00%. In contrast, the 150 mg VAN dose improved the ease of falling asleep by 51.18%, sleep quality by 36.5%, and morning wakefulness by 7.40%.

In comparison to the placebo, a single 150 mg VAN dose significantly improved the ease of falling asleep (*p* < 0.05), sleep quality (*p* < 0.05), and morning wakefulness (*p* < 0.05).

**Figure 7:**
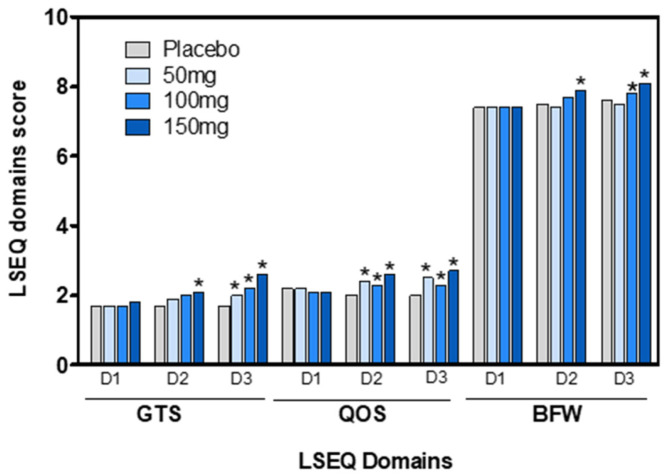
LSEQ score for domains GTS, QOS, and BFW obtained from the placebo or VAN-treated groups on days 1 (D1), 2 (D2), and 3 (D3). Significant differences between the placebo and 50, 100, or 150 mg VAN-treated groups were determined on days 1, 2, or 3 (* *p* < 0.05) with the ANOVA test.

Additionally, the lowest VAN dose (50 mg) significantly improved the sleep quality score (QOS) compared to the placebo-treated group 24 h after the start of treatment (day 2; Figure 7).

#### 2.3.4. Blood Pressure

Bidirectional effects were observed on both systolic and diastolic pressure after three days of treatment. A significant reduction in systolic pressure was observed with the 100 mg (*p* < 0.001) and 150 mg (*p* < 0.01) VAN doses, while a significant increase was observed with the 50 mg (*p* < 0.001) VAN dose. No significant changes were observed in the placebo group (Table 5).

For diastolic blood pressure, a significant reduction was observed in both the placebo group (*p* < 0.001) and the 100 mg VAN-treated group (*p* < 0.001). In contrast, a significant increase was noted with the 50 mg VAN dose (*p* < 0.001), while no significant changes were detected with the 150 mg dose. These changes in blood pressure appear to be related to a reduction in stress levels rather than through a direct physiological modulation of the cardiovascular system.

#### 2.3.5. Safety Result

All the randomized subjects were included in the safety analysis. Throughout the trial, no clinically significant differences in vital signs, physical examination results, or hematological and biochemical parameters were observed in any group. None of the subjects reported a serious adverse event (SAE) or were withdrawn from the study due to an adverse event (AE) or SAE.

Although a significant difference in cortisol levels was observed after treatment with the placebo or a 50 mg VAN dose in comparison to baseline (day 1), no significant differences were observed for the two groups treated with the highest doses of 100 or 150 mg. No changes in interleukin 1 (IL-1) and interleukin 6 (IL-6) levels were observed for any of the VAN doses tested, including interleukin 8 (IL-8) and tumor necrosis factor (TNF) at the 150 mg dose. However, small increases in the levels of both cytokines were observed for the VAN doses at 50 and 100 mg, although these were not statistically significant when compared to using the ANOVA test. The levels of these inflammatory biomarkers are shown in Supplementary Materials (Table of biochemicals variables, File S2). 

## 3. Discussion

Anxiety and stress are closely related psychological conditions that frequently co-occur and influence one another. Chronic stress can lead to the development of anxiety disorders, the dysregulation of the body’s stress response system, and alterations in hormones and neurochemicals, significantly affecting mood or sleep patterns. Furthermore, chronic stress can impair sleep by causing difficulties in falling asleep, maintaining sleep, or achieving restorative rest. In turn, poor rest or sleep deprivation results in daytime fatigue, irritability, and cognitive impairment during the day, which can amplify the perception of stressors. Therefore, it is critical to interrupt this negative feedback loop (stress–mood–sleep cycle) to restore overall well-being.

This study investigated the effect of VAN, standardized to 10% total vanilloids, on anxiety, emotional well-being, and sleep quality using the m-HAMA, POMS, and LSEQ questionnaires in a healthy adult population with moderate anxiety indicative of chronic stress. After three days of daily supplementation compared to the placebo, VAN doses ranging from 50 to 150 mg significantly improved the subjective perception of anxiety, mood, and stress, as well as sleep onset and quality, with no reported side effects. These results indicate that VAN may help restore the stress–mood–sleep cycle.

To the best of our knowledge, this is the first study to show that the *A. melegueta* seed extract has a beneficial effect on anxiety, stress management, mood, and sleep. The extract’s anxiolytic properties manifest rapidly, making it a promising supplement for mild anxiety, particularly due to its effectiveness within a short time frame and its absence of adverse effects. These biological effects highlight VAN’s potential to support the emotional well-being of individuals exposed to demanding lifestyles, including workers in high-pressure jobs, women going through perimenopause, and other populations dealing with daily challenges that may impact their emotional state.

Most of the health benefits attributed to *A. melegueta* have been associated with its anti-inflammatory and analgesic properties, primarily through the inhibition of Cyclooxygenase-2 (COX-2), leading to a concomitant decrease in prostaglandin synthesis [6,19]. This mechanism may help explain the positive effects of this spice in alleviating chronic inflammatory diseases [20]. In addition, a previous clinical trial demonstrated that 12 weeks of *A. melegueta* seed extract intake increased energy expenditure through the activation of brown adipose tissue (BAT) and a decrease in fat reduction [21,22]. In an animal model, the oral administration of 6-paradol significantly reduced visceral and subcutaneous fat depots, whereas no such effect was observed with 6-gingerol or 6-shogaol, suggesting that 6-paradol regulates several obesity-related genes in an AMPK-independent manner [23].

Notably, these three bioactive compounds—6-paradol, 6-gingerol, and 6-shogaol—are vanilloids, which are pungent components with activity on the TRPV1, similar to that of capsaicin. Animal studies have demonstrated that the oral administration of capsaicin can activate the TRPV1 expressed in sensory nerves within the gastrointestinal tract, increasing sympathetic nerve activity, which innervates the BAT. This results in a rapid rise in BAT temperature, increased whole-body energy expenditure (EE), and a reduction in body fat mass [24]. This connection suggests that the effects of vanilloids through TRPV1 activation could also be responsible for the increases in whole-body EE and reductions in body fat observed in clinical trials.

The in vitro results presented in this research show that VAN activates the TRPV1 in the same way as capsaicin, which correlates with the presence of vanilloid compounds present in VAN that are also responsible for the characteristic pungent taste of this species. Beyond the potential role of TRP1V in fat tissue, the therapeutic potential of TRP1V activation has primarily focused on the development of receptor antagonists as a source of analgesia [25]. However, the over-activation of the TRP1V can lead to desensitization, which may reduce tissue damage and inflammation [26]. This opens up the possibility of exploring VAN’s role as an analgesic in physiological situations that generate inflammation, although this was not the focus of the present study.

In a preclinical study, the administration of the *A. melegueta* seed extract to conscious, freely moving rats showed EEG modulation with prominent alpha-wave activity, indicative of the potentiation of serotoninergic signaling, similar to several antidepressants [16]. Serotonin modulation affects anxiety, depression, stress, memory, sleep, and thermal regulation and has been targeted by serotonergic antidepressants. The 5HTR1A activity is involved in these processes [27]. The 5HTR1A are widely distributed throughout the limbic system, cortical regions, and dorsal and median raphe nuclei and are expressed both pre- and post-synaptically. Both receptor elimination (knock-out models) and overexpression have been shown to lead to anxiety-like behaviors, suggesting a complex relationship between 5HTR1A activity and anxiety [28]. Several studies suggest that the activation of this receptor facilitates adaptation to stress [29], and animal models and clinical trials have demonstrated that both 5HTR1A agonists and partial agonists show promise in treating generalized anxiety disorder and depression [30].

The prominent alpha-wave activity reported in the electropharmacogram of rats administered the *A. melegueta* extract [16] is similar to that seen in rats treated with antidepressant drugs, underscoring the potential relationship between the *A. melegueta* extract and its functional application. The preliminary in vitro studies presented here show that VAN is capable of partially interacting with 5HTR1A. However, the neurophysiological relevance of this receptor modulation requires confirmation through specific functional in vivo tests. Although this partial modulation might not fully explain the alpha-wave activity observed in the in vivo EEG studies, previous research has reported functional crosstalk between the endocannabinoid and the serotoninergic system, involving various direct and indirect mechanisms across multiple brain regions (for review, see reference [31]). These interactions may underpin the parallel effects observed between the endocannabinoid and serotonin systems in the regulation of body temperature [32], feeding behavior [33], sleep and arousal [34], and emotional processes [35].

In recent years, anxiolytic treatments have increasingly focused on targeting the hCB1R, which is widely distributed across several brain regions, as well as in the peripheral tissues and organs, and is involved in regulating the balance between excitatory and inhibitory signals at the neuronal level [36]. However, the widespread distribution of hCB1R increases the likelihood that any therapeutic benefits may be accompanied by unwanted side effects. The endogenous hCB1R agonist anandamide (AEA), like 2-arachidonylglycerol (2-AG), is synthesized on demand and degraded by FAAH. Exposure to acute stress typically causes a rapid reduction in the tissue levels of AEA, which may be mediated, at least in part, by an increase in FAAH activity within the amygdala, hippocampus, or medial prefrontal cortex.

**Figure 8:**
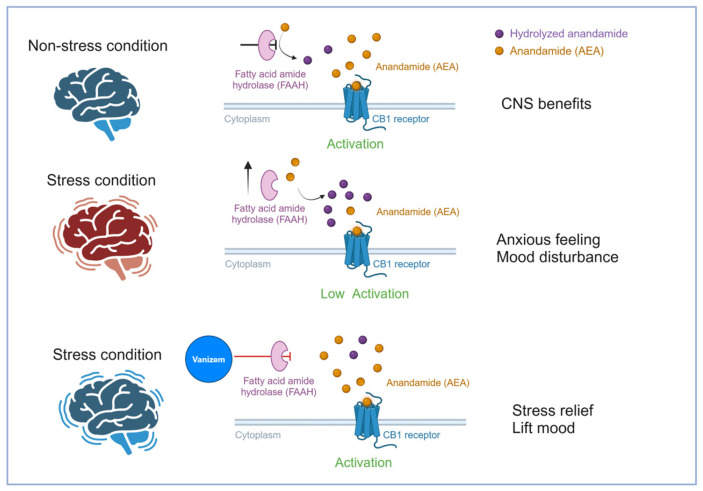
Proposed mechanism of action of *A. melegueta* seed extract.

The effects of stress on AEA levels appear to occur rapidly and may precede the activation of the HPA axis, implying that the underlying mechanism takes place before glucocorticoid release [37]. FAAH inhibitors are considered promising pharmacological targets for reversing the stress-induced anxiety state [38] with fewer side effects [39]. The in vitro results presented in this publication showed that VAN inhibits FAAH in a similar manner to URB597, which has been demonstrated to exert anxiolytic and antidepressant effects both in animals, models, and humans [40]. The inhibition of the FAAH enzyme suggests that VAN has a direct anxiolytic effect by restoring the buffering capacity of endocannabinoid signaling in the stress response (Figure 8). This mechanism may also explain the mood-elevating effects observed during the trial, as AEA is considered a mediator of emotional homeostasis [41].

In addition, VAN exerts the agonist modulation of hCB2R, which may contribute to its neuroprotective effect in situations of cognitive stress that can lead to neuroinflammation. hCB2R, present mostly in microglia and immune cells, is upregulated during tissue damage or neuroinflammation associated with psychiatric disorders, such as depression or anxiety. Its activation inhibits the release of proinflammatory mediators, resulting in a neuroprotective effect under neuropsychiatric conditions [42]. Moreover, findings from animal models suggest that hCB2R overexpression may mitigate behavioral responses to chronic stress [43]. It is hypothesized that, in conditions of cognitive dysregulation, the increase in immune system cells within the CNS, particularly the microglia, may account for the observed elevation in hCB2R, which may underlie the behavioral effects of hCB2R modulation [44].

This is the first study to demonstrate that the water–ethanolic extract of *A. melegueta* seed can inhibit enzymes and bind to receptors associated with the eCBs while providing clinical evidence for stress relief and mood enhancement. The immediate effects on sleep quality and sleep onset appear to result from the alleviation of daily stress. While the present study does not provide in vitro evidence to suggest that these sleep effects are mediated by the GABAergic or serotonergic systems, the modulation of eCB components by VAN may contribute to the breaking of the negative stress–mood–sleep cycle.

Several popular botanicals, through different mechanisms of action, have been shown to reduce anxiety and stress and improve mood, including *Crocus sativus* (saffron), *Piper methysticum* (kava kava), *Ginkgo biloba* (ginkgo), *Lavandula angustifolia* (Lavanda), and *Panax ginseng* (ginseng), although with limited evaluation of their effects on sleep [45]. In contrast, other plants used in several Western traditional systems of medicine, such as sleep aids, have been the subject of preclinical and human research as sedative agents in recent years to combat sleep disturbances. Among them, *Valeriana officinalis* (Valerian), *Passiflora edulis* (passionflower), *Matricaria chamomilla* (chamomilla), and *Melisa officinalis* (lemon balm) have shown the best potential for sleep support, primarily by modulating GABAergic neurotransmission in the brain [46,47]. However, although several clinical studies have shown remarkable positive results and generally favorable safety profiles, the quality of evidence differs substantially, and several concerns exist regarding the study design, purity grade, standardization, and potential negative herbal–drug interactions, especially with benzodiazepine (BZD) anxiolytic medication [48,49]. In addition, the roots of *Withania somnifera* (ashwagandha) are one of the most well-known and recognized ayurvedic ingredients for managing chronic stress, with significant improvements observed in symptoms of stress, anxiety, insomnia, or depression in human clinical trials. Noteworthy, its functional effects are mediated, almost in part, by the GABAergic system [48,49,50,51] and typically manifest after several weeks of consumption—at least 8 weeks—and at relatively high doses of 600 mg [52]. This contrasts with the present study, where significant improvements in sleep quality were observed with VAN from the first day, as well as improvements in stress and mood management after only three days at a lower dose (100–200 mg of extract) and apparently through a GABAergic-independent mechanism with potentially lower risks of anxiolytic drugs interactions.

From a safety viewpoint, vanilloid-rich extracts or seed preparations from *A. melegueta* are generally well tolerated in the medium-to-long term. In fact, except for one old uncontrolled clinical trial which showed that consumption of 0.35 g of *A. melegueta* seed resulted in blurred and double vision [53], all studies in humans evaluating the side effects of the supplementation of *A. melegueta* seeds for 4 to 12 weeks have shown no side effects at doses ranging from 30 to 500 mg/day of supercritical CO_2_ or ethanol extracts [21,22,54,55]. In addition, several clinical studies conducted with *Zingiber officinale* (ginger) rhizome powder or extracts, of which the main bioactive is vanilloids, with a duration of 2 to 28 weeks have reported no apparent side effects at doses between 1.2 and 2 g/Day [56,57,58].

Our study presents some limitations. First, this pilot trial included a small number of participants; hence, the extrapolation of the results to other populations should be made with caution. Second, the 3-day trial period was too short to provide data on long-term functional effects and/or the long-term safety risk associated with VAN. Third, the carry-over effects associated with the crossover design cannot be completely excluded. However, the counterbalance design, the progression strategy employed to exclude sequence effects, and the fast metabolization of VAN’s vanilloids linked to the 1-week washout period resulted in a negligible carry-over effect in the crossover study.

Future clinical studies should focus on a larger, more diverse population, an extended intervention duration, and parallel-group design to confirm these findings and evaluate VAN’s long-term safety in the organism. In addition, the use of selected rodent models to further investigate VAN’s mechanism of action at the CNS level and its potential synergistic effects with other botanical ingredients could shed light on its efficacy and applicability as a functional nutraceutical.

## 4. Materials and Methods

### 4.1. Chemicals and Reagent Used

Reference standards of 6-shogaol (Ref.: CDX-00019211-010), 6-gingerol (Ref.: CDX-00007164-010), 6-paradol (Ref.: CDX-00016068-050), and capsaicin (Ref.: CDX-00003135-010) were obtained from Chromadex (Los Angeles, CA, USA). The methanol and acetonitrile employed were of HPLC grade, and water was purified and deionized using a Milli-Q ultrapure water system (Millipore, Burlington, MA, USA).

### 4.2. Collection of Plant Material

The *A. melegueta* seeds used to prepare the botanical extract evaluated in this publication were obtained from local markets in the Eastern and Ashanti regions of Ghana, both located in the middle belt of the country. The ripe seeds were sun-dried at the time of purchase and stored under ambient conditions in the warehouses of Nektium Pharma S.L. until further use. To confirm the identity of the seeds as originating from the *A. melegueta* species, a specific DNA barcoding technique was performed at the Royal Botanical Garden (CSIC, Madrid, Spain) using DNA extracted from the seeds [59]. Briefly, DNA isolation from 500 mg of seeds in the batch was performed, and sequences from diagnostic chloroplast DNA matK were obtained. The matK region is used for the DNA identification of *Aframomum* and other *Zingiberaceae* species [59]. The analyzed sequence was compared to related sequences stored in GenBank using the BLAST search algorithm. The BLAST search retrieved accessions from the following species: *A. melegueta*, *A. daniellii*, *A. sceptrum*, *A. angustifolium,* and others. The phylogenetic analysis based on the ML algorithm grouped the obtained sequences with *A. melegueta* (See Supplementary Materials, File S3). Hence, the identity and phylogenetic analyses concluded that the matK sequence from the sample had an identity of 100% with the species *A. melegueta* K. Schum; therefore, the sample could be unequivocally identified as such.

### 4.3. Preparation of A. melegueta Seed Standardized Extract

**Figure 9:**
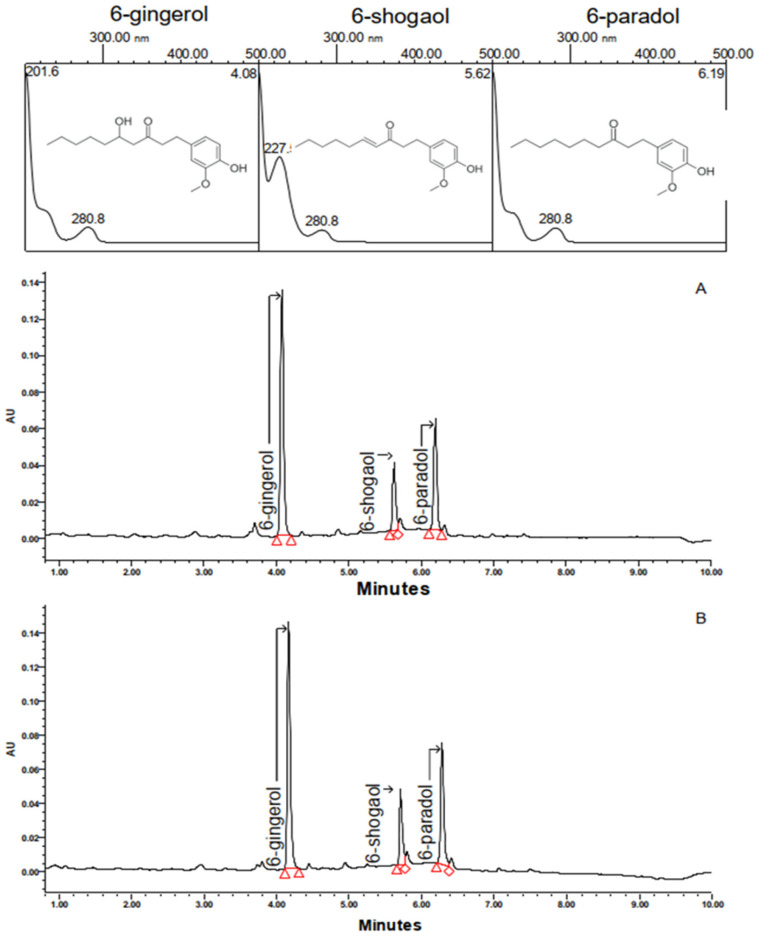
UV–vis spectra, chemical structures, and chromatographic profiles of *A. melegueta* seeds (**A**) and VAN (**B**) obtained at 280 nm.

The standardized vanilloid-rich extract of *A. melegueta* seeds tested in this study was manufactured by Nektium Pharma and is commercially available under the brand Vanizem^®^. The dried seeds were extracted using a mixture of ethanol and water (70:30, *v*/*v*). The native hydroalcoholic extract was then encapsulated by means of complex coacervation with a blend of gum arabic, pea protein, and low methyl-esterified citrus pectin. The colloidal solution containing the complex coacervate phase was spray-dried to produce a powdered extract. The final extract was standardized to contain no less than 10% (*w*/*w*) total vanilloids, as determined via ultra-high-performance liquid chromatography (UHPLC) with photodiode array (PDA) detection. The results are expressed as “total vanilloids”, which represents the sum of paradols, shogaols, gingerols, and related vanilloid compounds naturally present in the phytochemical fingerprint of *A. melegueta* as capsaicin equivalents. The total content of 6-paradol in VAN typically ranges from 1.5% to 5.5% (*w*/*w*), while shogaol ranges from 0.4% to 2.0% (*w*/*w*), and gingerol ranges from 2.5% to 7.0% (*w*/*w*). These ranges of concentrations are associated with the intrinsic variability of vanilloid-type compounds naturally occurring in *A. melegueta* seeds. The chromatographic profile and chemical structures of the major vanilloid compounds found in VAN are shown in Figure 9.

### 4.4. In Vitro Studies of A. melegueta Extract on Endocannabinoid System Targets

#### 4.4.1. Radioligand Binding Assay

A single concentration of VAN 200.3 μg/mL (20.03 μg/mL of total vanilloids) was tested to evaluate the percentage inhibition of control-specific binding across a panel of five receptors using a specific radiometric ligand-binding assay (Eurofins Cerep, Le Boisl Évêque, France). The receptors tested were as follows:

(1) The hCB2R—CB2 human cannabinoid GPCR binding agonist radioligand assay, code 37; (2) the human GABAAR-BZD—GABAA (alpha1/beta2/gamma2) human ion channel [3H] flunitrazepam binding agonist radioligand assay, code 4518; (3) the GABAAR—GABAA (alpha1/beta2/gamma2) human ion channel [3H] muscimol binding agonist radioligand assay, code 3051; (4) the 5HTR1A-5-HT1A human serotonin GPCR binding agonist radioligand assay, code 131; (5) the hCB1R—CB1 human cannabinoid GPCR binding antagonist radioligand assay, code 217050.

The results are expressed as the percentage of control-specific binding ((measured specific binding/control specific binding) × 100) obtained in the presence of the test material and as a percentage inhibition of control-specific binding ((100 − (measured specific binding/control specific binding)) × 100) obtained in the presence of the test materials.

Significant effects were defined as inhibitions (or stimulations for assays conducted under basal conditions) exceeding 75%. Results with inhibition (or stimulation) between 50 and 75% were considered indicative of moderate effects, and measurements showing inhibition (or stimulation) below 25% were not considered significant and were primarily attributed to variability around the control level.

#### 4.4.2. Cellular and Nuclear Receptor Functional Assay

A range of VAN concentrations at 100.1, 50, 25, and 2.5 μg/mL (calculated from the total vanilloid content) was tested to evaluate the percentage of agonist and/or antagonist responses against TRPV1. The functional bioassay employed was the TRPV1 Human Transient Potential Ion Channel Cell-Based Agonist and Antagonist Calcium Flux Assay, Code G106 (Eurofins Cerep SA, Le Boisl Évêque, France). TRPV1 activity (human recombinant expressed in CHO-S cells) was evaluated in the presence of reference compounds, including capsaicin (1 μM) as an agonist and capsazepine (240 nM) as an antagonist, as well as the four decreasing VAN concentrations described above. Intracellular [Ca^2+^] levels were measured by means of fluorometry following incubation at room temperature.

The results are expressed as the percentage of the control agonist response or inverse agonist response ((measured response/control response) × 100) and as the percentage inhibition of the control agonist response ((100 − (measured response/control response)) × 100) in the presence of the test materials.

#### 4.4.3. Enzyme and Uptake Assays

A single concentration of VAN of 1 × 10^−4^ M (calculated from total vanilloids; equivalent to 20.03 μg/mL of the VAN dry extract) was tested to determine the percentage of inhibition of FAAH using the standardized Fatty Acid Amide Hydrolase Human Enzymatic Assay, Code 199008 (Eurofins Panlabs Discovery Services Taiwan Ltd., New Taipei City, Taiwan). The recombinant FAAH, expressed in Sf21 cells, was tested alongside the reference inhibitory control compound of carbamic acid (URB-597), with an IC50 of 0.026 μM and VAN (1 × 10^−4^ M). The production of 7-amino-4-methyl coumarin from FAAH hydrolysis of the synthetic substrate AMC arachidonoyl amide was measured by means of spectrofluorometric quantification after 60 min of incubation at 37 °C. DMSO (1%) was used as the vehicle control.

The results are expressed as a percentage of the vehicle control response ((measured response/vehicle control response) × 100) and as the percentage of inhibition of the vehicle control response ((100 − (measured response/vehicle control response)) × 100) in the presence of the test material. Inhibition or stimulation higher than 75% was considered a “hit”, indicating the potential physiological effect of the test compound on the target and warranting further dose–response testing. Results showing inhibition (or stimulation) between 50 and 75% were indicative of moderate activity, while stimulation (or inhibition) between 25 and 50% was indicative of weak activity. Results with inhibition (or stimulation) below 25% were not considered active and were mostly attributed to signal variability around the background level.

### 4.5. Randomized Clinical Trial

#### 4.5.1. Study Design and Procedures

This clinical study was approved by the Ethics Committee of Kinetic Performance SL (Scientific Park, University of Alicante, Spain) under reference number K04/023 (on 9 January 2023). This clinical study was conducted in accordance with the Declaration of Helsinki and adhered to the International Good Clinical Practice (GCP) standards established by CPMP/ICH/135/95 (https://www.ema.europa.eu/en/documents/scientific-guideline/ich-guideline-good-clinical-practice-e6r2-step-5-revision-2_en.pdf, accessed on 13 February 2025) [60]. The trial was registered on Clinicaltrial.gov (NCT06463145). All participants provided written informed consent, which was securely stored and accessible only to authorized investigators. The detailed clinical protocol is freely available as a part of Supplementary Materials, Study protocol of the Spanish Ethics Committee, File S4 and Study protocol of the Spanish Ethics Committee (in Spanish), File S5.

Recruitment was performed using an information sheet, making targeted calls to eligible patients, and using the chain recruitment method in which participants referred to potential candidates. Adult males and females aged between 40 and 50 years with moderate anxiety, assessed using the modified Hamilton Anxiety Rating Scale (m-HAM-A; score range: 18–24), were invited to participate in the study. Participants attended their first visit at the GanaSalud Therapeutic Exercise Centre (Alicante, Spain), which included a screening session (day 0) to determine their eligibility based on the inclusion criterion, specifically, moderate anxiety as measured using the m-HAM-A. Additionally, to assess sleep quality before the start of the study, participants were classified as good or poor sleepers using the Pittsburgh Sleep Quality Index [61].

The exclusion criteria included a score greater than 20 on the Hamilton Depression Rating Scale (HDRS); prescribed treatments for anxiety, stress, or depression, as well as any medication that could affect sleep parameters or the activity of the sympathetic or parasympathetic nervous system; a history of drug or alcohol dependence; severe personality disorders that could interfere with study participation (psychosis, severe suicidal ideation); an intention to become pregnant (in female participants); epileptic disorders; liver disorders; a professional athletic status or engagement in extreme physical activity; and any external factors that could prevent the completion of the intervention. Eligible participants provided demographic and lifestyle data, along with baseline measurements of height, weight, waist-to-hip ratio, and blood pressure.

All participants received four formulations: 0 mg of VAN (placebo), 50 mg of VAN (dose A), 100 mg of VAN (dose B), and 150 mg of VAN (dose C). Each formulation was completed with maltodextrin as a filler so that all opaque capsules were identical in color, weight, and appearance to maintain the blinding of the study treatment. The study followed a randomized, monocentric, double-blind, placebo-controlled, crossover pilot trial design with two arms. Participants were randomly allocated to one of two groups with different treatment sequences: the ramp-up group (Group A), in which the order of treatments was placebo (0 mg), dose A (50 mg), dose B (100 mg), and dose C (150 mg), and the ramp-down group (Group B), in which the order was reversed—dose C (150 mg), dose B (100 mg), dose A (50 mg), and placebo (0 mg).

There was a seven-day washout period between each intervention. Over a three-day period, participants took one capsule in the morning before breakfast. On the first day (day 1), the capsules were taken at the study center, while on the following days (days 2 and 3), they were taken at home.

During the three-day intervention, participants were instructed not to take any remedies for anxiety, stress, or depression nor to consume two or more alcoholic drinks per day. As participants were routinely engaged in physical exercise, they were instructed to maintain their daily lifestyle habits with a standardized training program throughout the 33 days of the study.

The physical activity program included strength training sessions performed at 70–85% of one repetition maximum (1-RM), with a rating of perceived exertion (RPE) between 6 and 8 and a repetition in reserve (RIR) of 2–3. The exercises were multi-joint movements, including bench presses, high-pulley pull-downs, rowing, military presses, leg presses, heel raises, lateral raises, bicep curls, triceps extensions, and abdominal crunches.

Cardiovascular training consisted of 10–15 min of moderate physical activity, performed in 30 s to 1 min intervals on a bicycle. A second resistance training session was added for participants with a higher training volume prior to the study. Active participation during the study met the minimum recommendations for the development and maintenance of cardiovascular and musculoskeletal fitness. The training volume-load was calculated using the formula proposed by Cunha et al. (2020) [62]. All the training sessions were supervised by a graduate in physical activity and sports sciences, who adjusted the exercise loads of each exercise according to each participant’s ability to maintain the proper technique while performing the programmed resistance exercises. 

#### 4.5.2. Safety and Efficacy Parameters

The efficacy parameters utilized included questionnaire-based assessments: m-HAM-A for the anxiety evaluation, the Short-Form Profile of Mood States (SF-POMS) for emotional profile testing, and the Leeds Sleep Evaluation Questionnaire (LSEQ) for sleep evaluation. Safety parameters included physical examinations (weight and body composition), the measurement of vital signs (blood pressure, heart rate variability), and hematology and clinical chemistry tests (aspartate aminotransferase, alanine transaminase, serum creatinine, cortisol, and proinflammatory markers). Adverse events (AEs) and serious adverse events (SAEs) were monitored throughout the study period.

The HAM-A was modified in this study to assess symptom frequency. The HAM-A is a widely recognized psychometric scale for evaluating anxiety, consisting of 14 items that measure the severity of both psychic and somatic anxiety symptoms [63]. It is commonly used for both clinical and research purposes. In this study, each item was scored based on symptom frequency, using a scale of 0 (never) to 4 (always), resulting in a total score ranging from 0 to 56. A score between 18 and 24 was considered indicative of a moderate frequency of perceived anxiety in the m-HAM-A. Measurements were taken on days 1 and 3.

The Profile of Mood States (POMS) is a widely used 65-item self-report questionnaire to assess an individual’s mood and was employed in this study to evaluate participants’ stress levels and emotional responses [64]. Given the physical activity of the participants, an athlete-specific version of the POMS was employed. A shortened version of the POMS with 38 items (SF-POMS) was used and grouped into four dimensions according to Castañeda (2020): tension, fatigue, depression, and vigor [65]. Total mood disturbance (TMD), which provides an overall measure of emotional distress or disturbance, was calculated by summing the scores of three mood dimensions (tension + fatigue + depression) and subtracting the score for vigor. Measurements were taken on days 1 and 3.

The Leeds Sleep Evaluation Questionnaire (LSEQ) is a self-report tool used to assess various aspects of sleep quality and patterns [66]. This questionnaire includes items related to sleep onset latency, wakefulness after sleep onset, total sleep time, sleep efficiency, and overall sleep quality, grouped into the following four domains: Getting to Sleep (GTS), Sleep Quality (QOS), Episodes of Awakening from Sleep (AFS), and Behavior Following Wakefulness (BFW). Measurements were taken on days 1, 2, and 3.

#### 4.5.3. Statistical Analyses

Statistical analyses were performed using R software (v4.2.3). Continuous variables are presented as the means and standard deviation (SD). The normality of the data was assessed using the Shapiro–Wilk test. Paired comparisons of means were performed using the two-tailed Student’s *t*-test for normally distributed data or the Wilcoxon signed-rank test when the normality assumption was not satisfied. Repeated measures of ANOVA (for data with normal distribution) or the Friedman test (for data with non-normal distribution), with appropriate Bonferroni post hoc tests, were used to compare the differences between treatments. Statistical significance was set at *p* < 0.05.

A priori power calculations estimated that a minimum of 30 participants per group would be sufficient to detect a medium effect size in subsequent statistical analyses (*t*-test = 0.53 and ANOVA = 0.25) with 80% statistical power (alpha = 0.05).

Both the placebo (0 mg) and VAN capsules (50, 100, and 150 mg) were identical in appearance to maintain study blinding. Investigators, study staff, participants, and statisticians were all blinded to the treatment assignments. All participants who signed informed consent to participate in the trial were randomized using block randomization with a fixed block size of 6, stratified by sex and treatment in a 1:1 ratio. This method ensured a balanced allocation of participants across treatment types and sex. The fixed block size refers to the fact that the block size should be divisible by the number of intervention treatments to ensure balanced distribution within each block.

## 5. Conclusions

This study found that, after three days of ingestion of VAN, positive results were observed in anxiety reduction, stress alleviation, mood elevation, and sleep quality improvement. These effects may help to effectively break the negative feedback loop in the stress–mood–sleep cycle. The results also revealed a dose-dependent enhancement in sleep quality, even with a single dose. Notably, these improvements in sleep quality were achieved without any adverse effects or sedative properties.

## Figures and Tables

**Table 1:** Demographic and anthropometric baseline characteristics of the subjects.

Variables	Women (Mean ± SD)	Men (Mean ± SD)	Total (Mean ± SD)
Age (years)	46.15 ± 3.26	46.53 ± 2.27	46.37 ± 2.70
Weight (kg)	65.08 ± 12.08	78.07± 11.07	72.44 ± 13.07
Height (m)	1.65 ± 0.07	1.77± 0.08	1.72 ± 0.10
BMI (kg/m^2^)	23.83 ± 3.44	24.93 ± 2.86	24.45 ± 3.12
Fat mass (kg)	18.42 ± 7.16	15.61 ± 5.46	16.83 ± 6.30
Muscle mass (kg)	24.46 ± 3.41	35.05 ± 6.21	30.46 ± 7.39

SD: standard deviation. BMI: body mass index. Values are presented as the mean ± standard deviation.

**Table 2:** Comparison of anxiety scores ^1^ within study groups on days 1 and 3, 48 h after intervention.

Interventions	Evaluation on Day 1 (Mean ± SD)	Evaluation on Day 3 (Mean ± SD)	*p*-Value *
Placebo	19.4 ± 1.72	19.9 ± 2.18	0.079
50 mg	19.5 ± 2.05	17.8 ± 2.55	<0.01
100 mg	19.4 ± 2.60	14.9 ± 2.75	<0.01
150 mg	17.9 ± 2.28	12.5 ± 2.27	<0.01

All values are expressed as the mean ± standard deviation (SD). * Significant differences within each group were determined by comparing the scores before and 48 h after intervention (*p* < 0.01) using the Wilcoxon signed-rank test or Friedman test. ^1^ The Hamilton Anxiety Rating Scale Score (modified by frequency): the total score ranges from 0 to 56, with lower scores representing decreased anxiety levels.

**Table 3:** Profile of Mood States (POMS) scores within the study groups on day 1 and after 48 h of VAN treatment (day 3).

DMS	Doses	Evaluation on Day 1 (Mean ± SD)	Evaluation on Day 3 (Mean ± SD)	*p*-Value *
Tension	Placebo	8.57 ± 3.14	7.57 ± 3.24	<0.001
	50 mg	8.00 ± 3.14	7.53 ± 2.80	<0.05
	100 mg	7.50 ± 3.05	6.80 ± 2.66	<0.005
	150 mg	7.20 ± 3.13	6.07 ± 2.13	<0.005
Fatigue	Placebo	1.90 ± 2.20	1.97 ± 1.25	0.20
	50 mg	2.13 ± 1.36	1.97 ± 1.35	<0.05
	100 mg	2.00 ± 1.34	1.70 ± 1.35	<0.05
	150 mg	2.03 ± 1.30	1.50 ± 1.20	<0.01
Depression	Placebo	8.07 ± 3.77	8.17 ± 3.99	0.60
	50 mg	8.27 ± 3.84	7.07 ± 3.61	<0.01
	100 mg	7.73 ± 3.50	5.90 ± 2.89	<0.01
	150 mg	8.17 ± 3.47	5.33 ± 2.86	<0.01
Vigor	Placebo	10.40 ± 1.81	10.43 ± 1.74	0.30
	50 mg	10.50 ± 1.76	10.50 ± 1.61	1.00
	100 mg	10.37 ± 1.81	11.07 ± 1.41	<0.01
	150 mg	10.37 ± 1.69	11.27 ± 1.17	<0.01
TMD	Placebo	8.13 ± 5.22	7.27 ± 4.95	<0.01
	50 mg	7.90 ± 5.43	6.07 ± 5.24	<0.01
	100 mg	6.87 ± 4.42	3.33 ± 4.28	<0.01
	150 mg	7.03 ± 4.72	1.63 ± 3.81	<0.01

DMS: dimensions, TMD: total mood disturbance. Data are presented as the mean ± standard deviation. * Significant differences within each group were determined by comparing scores before and 48 h after intervention (*p* < 0.01 or <0.05), based on the Wilcoxon signed-rank test or Friedman test.

**Table 4:** Leeds Sleep Evaluation Questionnaire (LSEQ) scores within study groups before and after 48 h of VAN intervention (day 3).

DMS	Doses	Evaluation on Day 1 (Mean ± SD)	Evaluation on Day 3 (Mean ± SD)	*p*-Value *
GTS	Placebo	1.73 ± 0.52	1.70 ± 0.60	<0.77
	50 mg	1.70 ± 0.47	2.03 ± 0.56	<0.05
	100 mg	1.73 ± 0.58	2.23 ± 0.63	<0.01
	150 mg	1.80 ± 0.85	2.57 ± 0.63	<0.01
QOS	Placebo	2.20 ± 0.85	2.00 ± 0.69	0.12
	50 mg	2.17 ± 0.65	2.50 ± 0.57	<0.01
	100 mg	2.07 ± 0.64	2.73 ± 0.69	<0.01
	150 mg	2.10 ± 0.55	2.73 ± 0.52	<0.01
AFS	Placebo	1.97 ± 0.49	2.02 ± 0.48	<0.05
	50 mg	1.87 ± 0.43	2.07 ± 0.52	<0.05
	100 mg	1.80 ± 0.41	2.40 ± 0.62	<0.01
	150 mg	1.83 ± 0.46	2.33 ± 0.56	<0.01
BFW	Placebo	7.37 ± 1.81	7.57 ± 1.01	<0.71
	50 mg	7.40 ± 1.07	7.47 ± 1.01	<0.63
	100 mg	7.43 ± 1.04	7.83 ± 1.18	<0.05
	150 mg	7.73 ± 1.22	8.13 ± 0.82	<0.01

DMS: dimensions; GTS: ease of Getting to Sleep; QOS: perceived Quality of Sleep; AFS: Ease of Awakening from Sleep; BFW: integrity of Behavior Following Wakefulness. Data are presented as the mean ± standard deviation. * Significant differences within each group were obtained by comparing scores before and 48 h after intervention (*p* < 0.01 or <0.05) based on the Wilcoxon signed-rank test or Friedman test.

**Table 5:** Changes in blood pressure within each study group.

	Doses	Evaluation on Day 1 (Mean ± SD)	Evaluation on Day 3 (Mean ± SD)	*p*-Value *
Systolic	Placebo	116.50 ± 13.95	117.23 ± 14.78	ns
	50 mg	118.30 ± 14.15	122.67 ± 13.57	<0.001
	100 mg	120.87 ± 14.22	116.37 ± 14.45	<0.001
	150 mg	123.0 ± 10.97	119.87 ± 11.16	<0.01
Diastolic	Placebo	75.33 ± 8.76	72.97 ± 9.31	<0.001
	50 mg	78.20 ± 9.11	79.07 ± 8.52	<0.001
	100 mg	83.27 ± 8.80	79.33 ± 7.61	<0.001
	150 mg	82.27 ± 8.11	80.80 ± 8.68	ns

Data are presented as the mean ± standard deviation. * Significant differences within each group were determined by comparing the values before and 48 h after intervention using ANOVA (*p* < 0.01 or <0.001). “ns” indicates non-significant differences.

## Data Availability

All data matrix files of RCT are available from the OSF platform belonging to the Center for Open Science (https://osf.io/fv9bt, accessed on 5 August 2024). The other datasets used and/or analyzed during the current study are available from the corresponding author upon reasonable request.

## Supplementary Materials

The following supporting information can be downloaded at https://www.mdpi.com/article/10.3390/ph18020278/s1. File S1: Consort checklist; File S2: Table of biochemical variables; File S3: Phylogenetic identification; File S4: Study protocol of the Spanish Ethics Committee; File S5: Study protocol of the Spanish Ethics Committee (in Spanish).
