# Supplementary material for: Aframomum melegueta Seed Extract’s Effects on Anxiety, Stress, Mood, and Sleep: A Randomized, Double-Blind, Pilot Clinical Trial
Source: Pharmaceuticals (Basel). 2025 Feb 19;18(2):278. doi: 10.3390/ph18020278 (PMC11859572; doi:10.3390/ph18020278)
Supplement: Supplementary file 1 [file pharmaceuticals-18-00278-s001.zip › S2 File. Table of biochemical variables.pdf]

Biochemical variables				
	Doses (mg)	Day 1 Mean±SD	Day 3 Mean±SD	P
Cortisol (mg/dl)	0	10,76±3,39	11,26±3,13	<0,05
	50	10,83±3,59	11,22±3,87	<0,05
	100	10,46±3,39	10,40±3,52	n.s
	150	10,96±3,00	10,57±3,33	n.s
IL-1 (pg/mL)	0	1,98±3,94	1,84±3,44	n.s
	50	1,94±3,61	1,84±3,93	n.s
	100	2,01±3,36	1,81±3,20	n.s
	150	2,38±3,90	2,18±3,86	n.s
IL-6 (pg/mL)	0	2,30±0,66	2,08±0,21	n.s
	50	2,36±0,91	2,12±0,49	n.s
	100	2,12±0,46	2,11±0,43	n.s
	150	2,19±0,54	2,13±0,58	n.s
IL-8 (pg/mL)	0	131,25±59,41	150,1±60,05	<0,01
	50	133,56±58,33	146,42±66,28	<0,05
	100	143,23±56,49	156,45±66,22	<0,05
	150	138,59±57,38	134,73±56,8	n.s
TNF-α (pg/mL)	0	5,01±1,46	5,79±1,79	<0,01
	50	5,15±1,69	5,55±1,90	<0,05
	100	5,70±2,04	6,29±2,54	<0,05
	150	5,38±1,77	5,22±1,68	n.s
