# Supplementary material for: Aframomum melegueta Seed Extract’s Effects on Anxiety, Stress, Mood, and Sleep: A Randomized, Double-Blind, Pilot Clinical Trial
Source: Pharmaceuticals (Basel). 2025 Feb 19;18(2):278. doi: 10.3390/ph18020278 (PMC11859572; doi:10.3390/ph18020278)
Supplement: Supplementary file 1 [file pharmaceuticals-18-00278-s001.zip › S3 File. Phylogenetic identification.pdf]

Table S1. DNA isolation results

Sample	[DNA] (ng/µl)	Ratio 260nm/230nm	Ratio 260nm/280nm	PCR result
AF02	453.0	0.93	0.96	+++

Table S2. Average amount (%) of nucleotide identity between matK aligned sequence (PI parameter) and the closest species

Taxon Sample	*Length (bp)*	*Aframomum melegueta*	*Aframomum daniellii*	*Aframomum sceptrum*	*Aframomum angustifolium*
AF02	930	100	98.60	98.47	98.47
