# Supplementary material for: Aframomum melegueta Seed Extract’s Effects on Anxiety, Stress, Mood, and Sleep: A Randomized, Double-Blind, Pilot Clinical Trial
Source: Pharmaceuticals (Basel). 2025 Feb 19;18(2):278. doi: 10.3390/ph18020278 (PMC11859572; doi:10.3390/ph18020278)
Supplement: Supplementary file 1 [file pharmaceuticals-18-00278-s001.zip › S4 File. Study protocol Spanish Committe.pdf]

Effects of a plant extract modulating the endocannabinoid system and its anxiolytic capacity on senior people in situations of stress or anxiety.

PROTOCOL

PROMOTER OF THE STUDY

Nektium Pharma S.L.

C/ Las Mimosas 8, Polígono Industrial de Arinaga, 35118, Agüimes, las Palmas

Canary Islands, Spain

COMPANY/STUDY RESEARCH GROUP

Kinetic Performance S.L.

New Institutes of the Science Park of the University of Alicante, Carretera de San Vicente s/n 03690, San Vicente, Alicante, Spain

PRINCIPAL INVESTIGATOR OF THE STUDY

Dr. D. Carlos Elvira Aranda

Head of Research Kinetic Performance S.L.

New Institutes of the Science Park of the University of Alicante, Carretera de San Vicente s/n 03690, San Vicente del Raspeig (Alicante).

1. General information....................4
2. Objectives....................6
3. Design....................6
3.1. Description of the study design....................6
4. Selection of participants....................7
4.1. Inclusion criteria....................7
4.2. Exclusion criteria....................7
4.3. Withdrawal criteria....................7
5. Research treatment....................8
5.1. Description of treatment....................8
5.2. Allowed and prohibited medication....................8
5.3. Monitoring compliance....................8
6. Procedure and variables....................9
7. Timing and study visits....................11
8. Adverse events....................14
8.1. Definitions....................14
8.2. Procedure....................15
8.3. Reporting....................15
8.4. Information to Investigators....................15
9. Ethical aspects....................16
9.1. General considerations....................16
9.2. Patient information....................16
9.3. Confidentiality and access to data....................18
10. Practical considerations....................18
10.1. Responsibilities of all trial participants....................18
10.2. Deviations from the Protocol....................18
10.3. Protocol Amendments....................18
10.4. Investigator acceptance....................18
10.5. Commitment to publication of results....................18
11. References....................19
12. Annexes....................19

1. GENERAL INFORMATION

Research into the anxiolytic effect of a standardized plant extract that modulate the endocannabinoid system in senior people in situation of stress or anxiety.

Level of evidence	Date	Registration number CEI	Study code
Randomized Clinical Study	07/12/2022	K04/023	AME_HCT_2023

SPONSOR

Nektium Pharma S.L. (C/ Las Mimosas 8, Polígono Industrial de Arinaga, 35118, Agüimes, las Palmas)

STUDY TITLE

Effects of a plant extract modulating the endocannabinoid system and its anxiolytic capacity on senior people in situations of stress or anxiety.

PROTOCOLE CODE

AME_HCT_2023

RESEARCH TEAM

• Carlos Elvira Aranda (IP). Kinetic Performance S.L. Universidad de Alicante. GICAFD.

• Mario Terol Sanchis (Coord.). Kinetic Performance S.L. Universidad de Alicante. GICAFD. Centro de Ejercicio Terapéutico GanaSalud.

• Óscar Feltrer Hernández. Kinetic Performance S.L. Universidad de Alicante. GICAFD.

• Loreto Lledó Rico. Kinetic Performance S.L.

• Jaime Enrique Gómez Paternina. Kinetic Performance S.L.

• Francisco Bailén Gómez. Centro de Ejercicio Terapéutico GanaSalud.

• Rubén Acame Rocamora. Centro de Ejercicio Terapéutico GanaSalud.

• Sergio Pérez Galiana. Kinetic Performance S.L. Universidad de Alicante. GICAFD.

• Héctor Manuel Bernabé Lorca. Kinetic Performance S.L. Universidad de Alicante. GICAFD.

• José Palomares Baeza. Centro Inmunológico de Alicante (CIALAB) - Laboratorio Clínico de Ribera Salud (Riberalab).

CENTRES WHERE THE STUDY IS CONDUCTED

• Kinetic Performance S.L.

• Parque Científico de Alicante

• Universidad de Alicante

• Centro de Ejercicio Terapéutico GanaSalud

• Centro Inmunológico de Alicante (CIALAB) asociado a Ribera Salud como Laboratorio Clínico (Riberalab).

RESEARCH ETHIC COMITTE

Research Ethics Committee belonging to Kinetic Performance (Alicante Science Park).

2. OBJECTIVES

Main objective

To evaluate the anxiolytic effect of a plant seed extract that modulate endocannabinoid system in senior people experiencing anxiety.

Specific objectives

- To determine the effect of the product at different doses and its response on the anxiety dimension.

anxiety dimension.

- To analyze the changes in mood caused by the plant extract.

plant extract.

- To determine the influence of the plant extract on qualitative parameters of nocturnal

night-time sleep.

- To evaluate the sensitivity of heart rate variability parameters during night-time sleep.

heart rate variability during night-time sleep.

- To evaluate the physiological response through inflammatory markers during the different doses administered.

3. STUDY DESIGN

3.1. Description of the study design

The study will be conducted using a crossover experimental design with a randomized, double-blinded, cross-over comparison group. A single group will receive four doses of the product: 0mg, 50mg, 100mg and 150mg over a period of three days. Between each between each intervention there will be a seven-day washout period.

The dose application will be randomized under randomization codes and will follow two different profiles:

- Ramp up: 0mg - 50mg - 100mg - 150mg
- Ramp down: 150mg - 100mg - 50mg - 0mg

4. PARTICIPANTS

The group of participants will be composed of 37 elderly people (aged 40-50 years) who are experiencing a state of anxiety and who will be randomly assigned to receive the treatment dose.

4.1. Inclusion criteria

1. Age between 40 and 50 years.
2. Score above 18 points on the Hamilton Anxiety Scale (HAM-A).
3. Acceptance and signature of the informed consent for the study after having received adequate information.

4.2. Exclusion criteria

1. Score above 20 points on the Hamilton Depression Rating Scale (HDRS).
2. Receipt of medical treatment for anxiety, stress or depression.
3. Drug and alcohol dependence.
4. Severe personality disorders that may interfere with participation in the study (psychosis, ideation, severe suicidal ideation, etc.).
5. In the case of women, the intention to become pregnant.
6. Epileptic disorders.
7. Liver disorders (cirrhosis, hepatitis, etc.).
8. Professional sportsmen or those involved in extreme physical activities.
9. Inability to complete the intervention period due to external factors.

4.3. Withdrawal criteria.

All participants may be withdrawn if they meet any of the following withdrawal criteria at any time during the study:

1. Failure to meet any of the other inclusion criteria during the study.
2. Failure to meet any of the exclusion criteria during the study.
3. Product-related toxicity.
4. Failure to obtain the mandatory variables.

5. Failure to meet the established deadlines.

6. Loss of follow-up.

7. Death.

5. TREATMENT IN RESEARCH

5.1. Treatment description

All participants will receive four doses of the product: 0mg, 50mg, 100mg, 150mg. The intake protocol and washout period are detailed in the protocol design section. The product information is detailed in ANNEX 1.

5.2. Permitted and prohibited medication.

Any medication that does not affect the following is permitted:

- Medication prescribed for the treatment of anxiety, stress or depression, or any treatment that may affect these disorders.
- Alteration in sleep parameters.
- Alteration in the parasympathetic and sympathetic systems.
- Any drug or excessive alcohol intake is prohibited.

5.3. Compliance monitoring

The participant's compliance with the treatment will be assessed at each visit by the research staff.

6. PROCEDURE AND VARIABLES

The recruitment of candidates will be carried out by the research team in conjunction with the collaborating centers. The centers participating in this study are the technology-based company Kinetic Performance S.L., the University of Alicante, the Science Park of Alicante and the Therapeutic Exercise Centre GanaSalud. No specific material for participant recruitment, such as posters or advertisements was used. The participants will be selected among the professionals working in each center who are candidates for inclusion based on the specified inclusion and exclusion criteria.

All participants selected for possible inclusion will be scheduled for a first face-to-face screening visit with the participating physician and the principal investigator or the principal investigator/study coordinator, where their suitability for the study will be assessed against the inclusion and exclusion criteria, will be informed of the study and their written consent to participate will be sought.

The date of signature of the informed consent by the first participant shall be considered as the first act of selection.

The study protocol includes several procedures and variables to assess the effect of the plant extract on participants. These are the procedures and variables mentioned:

- **Anthropometric variables**: Day 0 (start of treatment) and +2 (end of treatment).

Measurement of body composition: Participants' age, height and weight will be measured. In addition, body composition will be assessed, including fat mass, percentage of fat mass, muscle mass, percentage of muscle mass and body mass index (BMI). These data will help to characterize participants and to take into account possible confounding factors related to body composition.

- **Blood pressure**: Day 0 (start of treatment) and +2 (end of treatment). Participants' blood pressure will be measured using an automatic blood pressure monitoring device. This measurement is important to assess the cardiovascular function of the participants and to detect possible changes related to the related to the plant extract intervention.

- **Biochemical analysis**: Day 0 (start of treatment) and +2 (end of treatment). Laboratory tests to measure various parameters will be carried to measure biochemical parameters in blood samples from participants. These tests will include complete blood count to assess the composition of blood cells. Levels of sodium, chlorine, magnesium and zinc, which are relevant minerals for metabolic and cellular functioning, will also be measured. In addition, GGT, GPT, GOT and FA enzymes, which are indicators of liver function, will also be tested.

Liver function. C-reactive protein, which is a marker of systemic inflammation, will be measured and pro-inflammatory cytokines IL-1, IL-6, IL-8 and TNF-α will be assessed.

Finally, basal serum cortisol, a hormone related to the stress response, will be measured.

- **Hamilton Anxiety Scale (HAM-A):** Day 0 (start of treatment) and +2 (end of treatment).

of treatment). APPENDIX 2.

- **Test Profile of Mood States (POMS):** Day 0 (start of treatment) and +2 (end of treatment).

treatment). APPENDIX 3.

- **Pittsburgh Sleep Quality Index (PSQI):** Day 0 (start of treatment) and +2 (end of treatment).

+2 (end of treatment). APPENDIX 4.

- **Leeds Sleep Evaluation Questionnaire (LSEQ):** Day 0 (start of treatment), +1 and +2 (end of treatment). APPENDIX 5.

- **Heart Rate Variability (HRV):** Day 0 (start of treatment), +1 and +2 (end of treatment). Heart rate variability shall be collected with POLAR H10+ heart rate bands during sleeping hours.

- **Patient diary:** Day 0 (start of treatment), +1 and +2 (end of treatment). Participants will be provided with a notebook in which they will record daily relevant information about their activities, food intake, the presence of symptoms such as headache or digestive problems. symptoms such as headache or digestive problems, as well as fatigue, and any other aspect that may be relevant to the study (APPENDIX 6). These records will provide subjective and detailed information about the participants' day-to-day life during the study.

Throughout the intervention period (detailed in section 7), participants will follow a standardized training programme, based on intensities, RPE or RIR:

- **Weekly volume**: 2-3 sessions x week (1:1 - Strength-endurance; 2:1 - Strength-endurance).
- **Session volume**: 1 hour (NOT LESS THAN 45 MINUTES).
- **Intensity**: 70-85% / RPE 6-8 / RIR 2-3
- **Density**: 1:2
- **Strength Training**: Prioritized multi-joint exercises. FullBody (No cluster. No Weider).
- **Cardiovascular Training**: Absolute intervals between 10-15' for RPE 7-8. Intervals between 30‘-1’. Prioritize in order bike, elliptical, treadmill.

7. STUDY SCHEDULE AND VISITS

The total duration of the study is estimated to be 18 weeks from the start of recruitment to the participant's last visit:

- Recruitment period: 8 weeks
- Treatment period: 10 weeks
- Individual study period: 33 days.

The visits of each patient in the study are carried out in the morning, complying with the time window from 7.00 to 10.30 am. Participants must attend in a fasting state, avoiding stimulating substances (e.g. coffee). The visiting procedure is detailed as follows:

Day 0. Start of treatment (Visit):

- Anamnesis.
- Blood pressure.
- Anthropometric variables
- Hamilton Anxiety Scale (HAM-A).
- Test Profile of Mood States (POMS).
- Pittsburgh Sleep Quality Index (PSQI).
- Leeds Sleep Evaluation Questionnaire (LSEQ).
- Blood sampling (biochemical variables).
- Heart rate variability* (during nocturnal sleep).
- Patient diary*.

Day +1. intervention (No visit):

- Leeds Sleep Evaluation Questionnaire (LSEQ).
- Heart rate variability (during nocturnal sleep).
- Patient diary.

*Variables are not carried out in the presence of the researcher.

Day +2. End of treatment (Visit):

- Anamnesis.
- Blood pressure.
- Anthropometric variables
- Hamilton Anxiety Scale (HAM-A).
- Test Profile of Mood States (POMS).
- Pittsburgh Sleep Quality Index (PSQI).
- Leeds Sleep Evaluation Questionnaire (LSEQ).
- Blood sampling (biochemical variables).
- Heart rate variability* (during nocturnal sleep).
- Patient diary.

Day +3 to +9. Washing period (No visit):

- Participant rest. Custody of heart rate strap or return of heart rate strap on these days if study ends

Tabla 1

Description of the intervention period

	DAY 0	DAY +1	DAY +2
	Start	Treatment	End
Anamnesis			
Vital constant			
Anthropometric measurements			
Analytics			
HAM-A			
POMS			
PSQI			
LSEQ			
VFC			
Patient diary			
Adverse events			
(1) Arterial pressure			

8. ADVERSE EVENTS

8.1. Definitions

Adverse Event (AE)

Any adverse health occurrence in a patient or clinical trial subject treated with a medicinal product, even if not necessarily causally related to the medicinal product.

AE means any untoward health occurrence in a patient or clinical trial subject treated with a medicinal product, even if not necessarily causally related to that treatment.

Adverse Reaction (AR)

An AR is any unintended, harmful reaction to an investigational medicinal product, regardless of the dose administered.

Serious Adverse Event (SAE) and Serious Adverse Reaction (SAR)

Serious SAE or SAR are considered to be those which, at any dose, may result in death, life-threatening, requiring hospitalization of the patient or prolonging an existing hospitalization, cause permanent or significant disability or incapacity, or result in an anomaly or malformation result in a congenital anomaly or malformation. Also considered serious are those medically significant suspected SAE or SAR, even if they do not meet the above criteria, are also considered serious, including medically significant events requiring intervention to prevent the occurrence of one of the consequences described above.

In addition, all suspected transmission of an infectious agent via a medicinal product shall be reported as serious. infectious agent via a medicinal product shall also be reported as serious. The term 'life-threatening' in the definition means that in the opinion of the definition refers to the fact that, in the opinion of the investigator, the patient at the time of the SAE or SAR is at real risk of death; and RA is at actual risk of death; it does not refer to the fact that the SAE/SAR hypothetically could have resulted in death had the AA/RA would have resulted in death had it been more severe.

Unexpected Adverse Reaction (UAR)

Any AR whose nature, severity or consequences do not correspond to the reference safety information.

Serious Unexpected Adverse Reaction (SUSAR)

SUSAR (previously defined), which nature, severity or consequences do not correspond to the reference safety information.

8.2. Procedure

The investigator will systematically collect and follow up AEs from the first administration of the investigational product until the final follow-up visit of each participant. All AE spontaneously reported by the participant and/or in response to an open-ended question by the investigator or those observed or objectified in the physical examination or in any complementary test will be recorded in the participant's CRD.

All SAEs experienced by a participant, regardless of their presumed causality, shall be monitored until the event has resolved or stabilized, the abnormal laboratory values that gave rise to the AR or SAEs have returned to baseline values or have stabilized to a level acceptable to the investigator, there is a satisfactory explanation for the observed changes, or if the monitoring of the patient could not be followed up. Adverse reactions will be monitored until resolution or stabilization (whichever occurs first).

The investigator shall assess and record in detail the AEs, including the start and end date, description of the event, severity, course, outcome, relationship of the AEs to the investigational medicinal product, and actions taken (treatments, additional complementary explorations, etc.).

The decision as to whether the intensity of an AE is sufficient to withdraw the patient from the study shall be made at the clinical judgement of the investigator. The patient may also decide to withdraw from the study at his or her discretion if he or she considers the intensity of the AE to be unbearable.

8.3. Notification

In the event of an SAE, the investigator shall notify the sponsor or the person assuming the tasks delegated by the sponsor within 24 hours of becoming aware of the SAE.

8.5. Information to researchers

The Principal Investigator (PI) will be responsible for managing the information related to this aspect between the sponsor and the research team.

9. ETHICAL ISSUES

9.1. General considerations

This trial should be conducted in accordance with the protocol and with the standards of good clinical practice as described in the current applicable legislation. The investigator team agrees to follow the instructions and procedures described in the protocol and will therefore comply with the principles of Good Clinical Practice on which it is based. Modifications to the protocol will be agreed upon by both the sponsor and the principal investigator, and with the consent of the Research Ethics Committee (REC).

9.2. Information to the patient

During the screening visit, the investigator is responsible for fully and thoroughly informing the participant of all relevant aspects of the trial, the nature and objectives of the trial, and the potential risks involved, including the written information and the favourable opinion of the ethics committee (APPENDIX 7). The language used in the oral information should not be technical but practical and should be understandable to the participant. In addition, the information should not contain any language that would lead to waiver or appearance of waiver of any legal rights, or that would release or appear to release the investigator, the trial site, the sponsor, or the sponsor's staff from their obligations in the event of negligence. Neither the investigator nor the trial staff should coerce or unduly influence the subject to participate in the trial. Specifically, the subject should be informed of the following details:

- That the study represents research.
- The purpose of the study.
- The study treatments and the probability of randomization for each treatment.
- The procedures to be followed in the study, including all invasive procedures.
- The participant's responsibilities.
- Reasonably foreseeable risks or inconveniences to the participant.
- That the participant's participation in the study is voluntary and that the participant may refuse to participate or withdraw from the study at any time.
- That the monitors, auditors, ethics committee and competent authorities will have direct access to the participant's original medical records for verification of study procedures or data, without violating the confidentiality of the participant, to the extent permitted by the relevant regulations and that, by signing the informed consent form, the participant or his/her legal representative is authorizing access to these data.

- That records identifying the participant will be kept confidential and, as permitted by applicable laws or regulations, will not be made publicly available. If the results of the study are published, the identity of the participant will be kept confidential.

- That the participant will be informed at all times if new information becomes available that may change the participant's decision to continue in the study.

- The foreseeable circumstances or reasons under which the participant's participation in the study may be terminated.

- The expected duration of the participant's participation in the study.

The investigator will submit an appropriately completed, written, GCP-compliant informed consent form in accordance with ICH guidelines and local legal requirements to the REC for review and approval prior to initiation of the trial (APPENDIX 8). Prior to trial participant entry, a copy of the REC-approved informed consent form will be reviewed with the potential participant, signed and dated. The investigator will provide a copy of each participant's signed informed consent form and retain a copy in the subject's study file.

9.3. Confidentiality and access to data

All information collected will be treated as strictly confidential, in accordance with current regulations (Regulation (EU) 2016/679 of the European Parliament and of the Council of 27 April 2016 (GDPR), Organic Law 3/2018 of 5 December on the Protection of Personal Data and Guarantee of Digital Rights Law 41/2002 on Patient Autonomy, General Health Law 14/1986 and Biomedical Research Law 14/2007).

The confidentiality of the subjects' personal data will be maintained, although subject to the need, on the part of the monitor, to verify the original data against the subject's clinical history. The electronic data collection notebook and all study correspondence will contain only the patient code, which will consist of a number indicating the center followed by a two-digit number to be assigned in order of inclusion.

The correspondence between the patient's identity and this code will be kept in a separate document and guarded by the research team. All information disclosed by the sponsor to the investigator will be treated as strictly confidential. The investigator will only use this information for the study described in this protocol. Furthermore, the investigator undertakes not to disclose this information to third parties, except to other colleagues or employees involved in the execution of the study who are also bound by confidentiality obligations.

10. PRACTICAL CONSIDERATIONS

10.1. Responsibility of All Trial Participants

- Investigator: Investigators should adhere to the standards of Good Clinical Practice and should be familiar with and follow the protocol procedures. All information collected during the conduct of the trial should be recorded directly in the data collection notebook. When a correction is made, the date and initials of the person making the correction should be noted.

- Auxiliary personnel: The auxiliary personnel shall follow the instructions given by the investigator regarding blood sampling, handling and other complementary examinations.

- Sponsor: Responsible for ensuring compliance with the relevant legal requirements and for supplying the study medication.

10.2. Deviations from the protocol

The investigator should not make any deviation or modification to the protocol without the sponsor's permission, and prior review and written approval of the modification by the Ethics Committee, except when it is necessary to reduce an imminent risk to the participants or when the modification involves only logistical or administrative aspects.

In such a case, any deviation from the approved protocol should be documented and explained by the investigator, or a person designated by the investigator, and submitted to the sponsor for concurrence as soon as possible.

10.3. Amendments to the protocol

No amendments to the protocol are recorded.

10.4. Investigator's acceptance

The investigator's agreement is included in the documentation submitted to the IRB.

10.5. Commitment to publish results

This is reflected in the model contract between Nektium Pharma S.L. and Kinetic Performance S.L.

11. REFERENCES

Specific references to the questionnaires, variables and procedures used in this study will be included in the final report.

12. ANNEXES

ANNEX 1. PRODUCT INFORMATION.

ANNEX 2. HAMILTON ANXIETY SCALE (HAM-A).

ANNEX 3. TEST PROFILE OF MOOD STATES (POMS).

ANNEX 4. PITTSBURGH SLEEP QUALITY INDEX (PSQI).

ANNEX 5. LEEDS SLEEP ASSESSMENT QUESTIONNAIRE (LSEQ).

ANNEX 6. PATIENT DIARY.

ANNEX 7. RESEARCH ETHICS COMMITTEE APPROVAL.

ANNEX 8. INFORMED CONSENT.

COD: AME_HCT_2023

ANEXE: *Aframomun melegueta*

RESPONSIBLE RESEARCHER AT NEKTIUM:
Dra. Laura López Ríos

EXTRAC OF AFRAMOMUM MELEGUETA

- 70% hydroalcoholic extract of seeds.
- Standardized to total gingerosides.
- Allergen declaration (Directive 2007/68/EC): Although no known allergens have been reported in the literature, possible allergic reactions in people sensitive to capsaicin or gingerols are not excluded.
- This product is not derived from genetically modified organisms (non-GMO).
- It is suitable for human consumption.

AFRAMOMUM MELEGUETA

Aframomum melegueta (AM) is an herbaceous species of the Zingiberaceae family, commonly known as ‘grains of paradise’ or ‘melegueta pepper’. It is an aromatic, perennial rhizomatous herbaceous plant whose seed is commonly used as a condiment or culinary spice.

TRADITIONAL USE OF THE SEED

Apart from their traditional culinary use (as a spice or added to drinks), grains of paradise have also been used in East Africa to treat colds and sore throats or to warm up on cold days (chewed). The chewed seeds or in decoctions have also been used to treat dysentery, abdominal pain, constipation, rheumatism, inflammation, snakebite and hypertension. Mixed with salt and placed inside the mouth, they have been used to treat sleep disturbances. In men, they have been used in combination with other plants to treat infertility. In animals, the seeds soaked in alcohol are given to hunting dogs as an excitant (Trinidad). (1)(2)(3).

NUTRITIONAL COMPOSITION

The vitamin with the highest concentration is vitamin C (12.3 mg/100g) but it also contains thiamine (Vit B1), Riboflavin and nicotinic acid. The mineral present in the highest concentration is iron (1.8 mg/100g) and it also contains magnesium, calcium, phosphorus, sodium, zinc, copper and manganese. It contains piperine, an alkaloid found mainly in black pepper, which gives it its bitter taste. (4).

The total polyphenol content varies from 2-2.8%, mainly tannins and flavonoids (such as quercetin, kaempferol and hydroxybenzoic acid). The most relevant active compounds in Aframomum melegueta fall into a group known as gingerols, which includes 6-gingerol (1-1.6%) followed by 6-paradol, 7-paradol and 6-shogaol. (4).

PROPERTIES AND SCIENTIFIC STUDIES

Both in vitro and in vivo studies have been carried out on its properties and it has been found to be a good antioxidant, 400 g of seed have a similar effect to vitamin E (5), its effect may be dose-dependent (6) and it could modify the response of white cells to tissue damage (7). It has anti-inflammatory capacity as it inhibits the production of prostaglandins and leukotrienes as well as nitric oxide synthase (NOS) and COX2 (Cyclooxygenase 2) (in vitro) (6) and the expression of pro-inflammatory genes (1). An anti-stress effect has also been observed by inhibiting beta-adrenergic receptors (8), (9), anti-estrogenic (10) and neuroprotective (in animal models of traumatic brain injury (TBI), an extract of *Aframomum melegueta* reduced neurodegeneration in TBI survivors and, in some cases, restored gene expression (11). A recent study in rats evaluated the beneficial effect of an alcoholic extract of AM on scopolamine-induced cognitive impairment (12).

At the level of metabolic homeostasis, the seed extract and 6-paradol (present in the alcoholic extract of the seeds), can increase brown fat temperature and reduce visceral fat, and is therefore considered a thermogenic (13). In mouse models of type 2 diabetes (treated with alloxan), blood glucose levels were lowered more in animals treated with an aqueous extract of the seeds than those treated with metformin (14). It also reduces cholesterol levels, ACE activity and pancreatic enzymes (hepatoprotective) in hypercholesterolemic rats and these effects could be modulated by its amino acid content (such as GABA, tyrosine, phenylalanine and tryptophan combined with the antioxidant phytochemicals it contains (15).

It exerts an anti-ulcer effect and increases gastric mucosal resistance (16). Both alcoholic and aqueous extracts can act as anti-fungal or anti-bacterial against gram-positive and gram-negative bacteria (e.g. E. coli, Pseudomonas, Shigella

spp. or Klebsiella) (17,16). And liver improvement has been observed in rats with induced liver damage following administration of alcoholic extracts or seed powder dissolved in water (18).

In male animal models, AM extract improved erection and increased both the volume and frequency of ejaculation (1,19), reduced PSA (Prostate Specific Antigen) levels and increased testosterone levels (20).

HUMAN CLINICAL TRIALS

Drug enhancer: In vitro studies have shown the ability of the aqueous extract and alcoholic extract of A. melegueta to inhibit CYP3A4, CYP3A5 and CYP3A7, thus interfering with the metabolism of some drugs (in vitro) (21).

Hypertension: Study in normotensive and hypertensive subjects (n=18) ingestion of 10-20 seeds (1.2mg/gran) of grains of paradise appeared to exert a positive effect on blood pressure levels in both normotensive and hypertensive subjects (22).

Total energy expenditure (EE): Brown fat imaging (PET) study with 19 young (male) healthy participants who were administered 40mg of alcoholic extract of AM. From the study it was concluded that AM extract increased brown adipose tissue activity leading to an increase in total energy expenditure. In another study, administration of 30 mg of MA extract to 19 young women for 4 weeks resulted in a reduction in abdominal waist circumference. In both studies, no side-effects or discomfort from taking MA extract were reported (23,24).

Eye condition: After a single dose of 350 mg of AM seeds was administered to 10 healthy, normal-weight men aged 30-35 years, an increase in the point of convergence (17.2%) and a reduction in the amplitude of acular accommodation (9.2%) were observed, resulting in transient double and blurred vision (25).

Quality of life in pre-menopausal women: After administration of a dose of 50mg/day, included in a combined formulation with two other ingredients, for 8 weeks, a total of 57 pre-menopausal women showed no side effects and an improvement in the score on the quality-of-life scale specific for this group.

SEGURIDAD Y TOXICIDAD

Acute phase studies: Administration to rats of an alcoholic extract of AM seeds at 0.25-4g/kg b.w. was not associated with toxic symptoms or mortality (14-day observation period) (16).

Chronic phase studies: After administration of an aqueous extract of MA, no histological changes in the adrenal gland were observed, although weight gain was observed. On the other hand, administration of an alcoholic extract of MA (26).

Hepatotoxicity: A study was conducted in rats administered a methanolic extract of 300 mg/kg body weight and sub-chronic exposure was assessed at 7, 14 and 21 days. Liver toxicity was assessed in relation to altered levels of circulating liver enzymes in serum. AST (aspartate aminotransaminase) levels progressively increased. ALT (alkaline phosphatase), ALP (alanine aminotransferase) and total bilirubin levels were slightly and significantly elevated at 14 and 21 days. Serum albumin levels were unchanged during this period. Liver histopathology revealed medium necrosis locally at 7 days, moderate multifocal at 14 days and severe at 21 days. Thus, at a dose of 300 mg/kg animal, the methanolic extract of AM induces liver toxicity (27). On the other hand, the aqueous extract or the 45% alcohol extract seems to have a beneficial effect on the liver (hepatoprotective) due to its antioxidant capacity at the hepatic level (18).

PREGNANCY AND CONCEPTION

Studies in rats have shown that intraperitoneal administration of an aqueous extract of the seeds reduced weight gain during pregnancy. Abortifacient effect is also attributed to high doses (4mg/kg body weight) (28).

It is therefore not advisable to administer it at high doses to fertile female populations ready to produce offspring.

REGULATORY STATUS

Aframomum melegueta seed is included as ‘food’ in the BelFrIt list (Harmonized list of food supplements of botanical origin agreed by Belgium, France and Italy).

The European Commission, in the Novel Food catalogue, considers the seeds fit for consumption as they were on the market as food or food ingredient and were largely consumed before 15 May 1997. Therefore, their access to the market is not subject to the Novel Food Regulation (EC) No 258/97.

Aframomum melegueta, also known as grains of paradise, is mentioned in the Code of Federal Regulations (FDA, USA) Title 21 ‘21 CFR - PART 182 - SUBSTANCES GENERALLY RECOGNIZED AS SAFE - Sec. 182.10, section identifying for human consumption spices and other flavourings. Furthermore, because it is listed in the United Natural Products Alliance, the Herbs of Commerce, first edition (1992) and the American Herbal Products Association's Botanical Safety Handbook (1997) it is considered as an ‘Old Dietary Ingredient’ and fit for human consumption.

The African Herbal Pharmacopoeia contains information describing the plant, part of its use, ethnopharmacological uses and its composition.

MARKET PRESENCE

PATENT

The European Patent Office lists at least three patents covering *Aframomum melegueta* for different applications.

FINAL PRODUCT

Parodoxine™ is a product based on grains of paradise extract standardized to 12-15% gingerols. It has been developed by GENABOLIX, aimed at fat burning and weight loss. A dose of 40mg/serving is recommended.

We can also find AM extract as part of the composition of products intended for sports to enhance fat burning and thermogenesis (Examples: After burner, from 'Magnum Nutraceutical' manufacturer and Diablo™ from 'ANS performance' company, etc) at doses of 40mg per capsule and where it is recommended to take three capsules per day.

It can also be found on the market as an unbranded generic standardized to different concentrations of 6-paradol (12-55%), both as a powder and as a liquid extract.

REFERENCES

1. Umukoro S, Ashorobi RB. Further studies on the antinociceptive action of aqueous seed extract of Aframomum melegueta. J Ethnopharmacol. 2007 Feb 12;109(3):501–4.
2. Iwu MM. Handbook of African Medicinal Plants. 1º ed. London: CRC Press; 1993. 506 p.
3. Lans C, Harper T, Georges K, Bridgewater E. Medicinal and ethnoveterinary remedies of hunters in Trinidad. BMC Complement Altern Med. 2001;1:10.
4. D.O. A, K.T. O, T.P. O, O.J. O, I.A A. Nutritional, Minerals and Phytochemicals composition of Garcinia cola [Bitter cola] and Aframomum melegueta [Alligator pepper]. J Environ Sci Toxicol Food Technol. 2014;8(1):8 6–91.
5. Adegoke GO, Makinde O, Falade KO, Uzo-Peters PI. Extraction and characterization of antioxidants from Aframomum melegueta and Xylopia aethiopica. Eur Food Res Technol. 2003 Jun 22;216(6):526–8.
6. Dugasani S, Pichika MR, Nadarajah VD, Balijepalli MK, Tandra S, Korlakunta JN. Comparative antioxidant and anti-inflammatory effects of [6]-gingerol, [8]-gingerol, [10]-gingerol and [6]-shogaol. J Ethnopharmacol. 2010 Feb 3;127(2):515–20.
7. Umukoro S, Ashorobi BR. Further pharmacological studies on aqueous seed extract of Aframomum melegueta in rats. J Ethnopharmacol. 2008 Feb 12;115(3):489–93.
8. Ahounou JF, Ouedraogo GG, Gbenou JD, Ouedraogo S, Agbodjogbe WK, Dansou PH, et al. Spasmolytic effects of aqueous extract of mixture from Aframomumum melegueta

(K Schum) - Citrus aurantifolia (Christm and Panzer) on isolated trachea from rat. African J Tradit Complement Altern Med AJTCAM. 2012;9(2):228–33.
9. Umukoro S, and Ashorobi R. Anti - Stress Po tential of Aqueous Seed Extract of Aframomum Melegueta. African J Biomed Res. 2005;8:11 9–1 21.
10. Ali M. El-Halawanya MH. Anti-oestrogenic diarylheptanoids from Aframomum melegueta with in silico oestrogen receptor alpha binding conformation similar to enterodiol and enterolactone. Food Chem. 2012;134(1):219–26.
11. Kumar A. Neuroprotective Effects of Aframomum melegueta Extract after Experimental Traumatic Brain Injury. Nat Prod Chem Res. 2015;3(1).
12. Ishola IO, Awoyemi AA, Afolayan GO. Involvement of Antioxidant System in the Amelioration of Scopolamine-Induced Memory Impairment by Grains of Paradise (Aframomum melegueta K. Schum.) Extract. Drug Res (Stuttg). 2016 Sep;66(9):455–63.
13. Iwami M, Mahmoud FA, Shiina T, Hirayama H, Shima T, Sugita J, et al. Extract of grains of paradise and its active principle 6-paradol trigger thermogenesis of brown adipose tissue in rats. Auton Neurosci. 2011 Apr 26;161(1-2):63–7.
14. Adesokan Ayoade A* AMA and AGS. Evaluation of Hypoglycaemic Efficacy of Aqueous Seed Extract of Aframomum melegueta in Alloxan-induced Diabetic Rats. Sierra Leone J Biomed Res. 2010;2(2):91–4.
15. Adefegha SA, Oboh G, Adefegha OM, Henle T. Alligator pepper/Grain of Paradise (Aframomum melegueta) modulates Angiotensin-I converting enzyme activity, lipid profile and oxidative imbalances in a rat model of hypercholesterolemia. Pathophysiol Off J Int Soc Pathophysiol. 2016 Sep;23(3):191–202.
16. S. Rafatullah, A. M. Galal MAA-Y& MSA-S. Gastric and duodenal ulcer and cytoprotective effects of Aframomum melegueta in rats. Int J Pharmacogn. 1995;33(4):311–6.
17. Konning GH, Agyare C, Ennison B. Antimicrobial activity of some medicinal plants from Ghana. Fitoterapia. 2004 Jan;75(1):65–7.
18. Nwozo SO, Oyinloye BE. Hepatoprotective effect of aqueous extract of Aframomum melegueta on ethanol-induced toxicity in rats. Acta Biochim Pol. 2011;58(3):355–8.
19. Mbongue GYF, Kamtchouing P, Dimo T. Effects of the aqueous extract of dry seeds of Aframomum melegueta on some parameters of the reproductive function of mature male rats. Andrologia. 2012 Feb;44(1):53–8.
20. Akpanabiatu MI, Ekpo ND, Ufot UF, Udoh NM, Akpan EJ, Etuk EU. Acute toxicity, biochemical and haematological study of Aframomum melegueta seed oil in male Wistar albino rats. J Ethnopharmacol. 2013 Nov 25;150(2):590–4.
21. Agbonon A, Eklu-Gadegbeku K, Aklikokou K, Gbeassor M, Akpagana K, Tam TW, et al. In vitro inhibitory effect of West African medicinal and food plants on human cytochrome P450 3A subfamily. J Ethnopharmacol. 2010 Mar 24;128(2):390–4.
22. . BASL, . AOA, . GAE, . ADE. Hypotensive and Antihypertensive Effects of Aframomum melegueta Seeds in Humans. Int J Pharmacol. 2007 Apr 1;3(4):311–8.
23. Sugita J, Yoneshiro T, Hatano T, Aita S, Ikemoto T, Uchiwa H, et al. Grains of paradise (Aframomum melegueta) extract activates brown adipose tissue and increases whole-body energy expenditure in men. Br J Nutr. 2013 Aug;110(4):733–8.
24. Sugita J, Yoneshiro T, Sugishima Y, Ikemoto T, Uchiwa H, Suzuki I, et al. Daily ingestion of grains of paradise (Aframomum melegueta) extract increases whole-body energy expenditure and decreases visceral fat in humans. J Nutr Sci Vitaminol (Tokyo). 2014;60(1):22–7.
25. Igwe SA, Emeruwa IC, Modie JA. Ocular toxicity of Afromomum melegueta (alligator pepper) on healthy Igbos of Nigeria. J Ethnopharmacol. 1999 Jun;65(3):203–6.
26. Obike H I, Ezejindu D N AA. The effects of Aframomum melegueta aqueous extract on the adrenal gland of adult wistar rats. Int J Res Med Heal Sci. 2014;3(6):1–6.
27. Ilic N, Schmidt BM, Poulev A, Raskin I. Toxicological evaluation of grains of paradise (Aframomum melegueta) [Roscoe] K. Schum. J Ethnopharmacol. 2010 Feb 3;127(2):352–6.
28. Inegbenebor U, Ebomoyi MI, Onyia KA, Amadi K, Aigbiremolen AE. Effect of aqueous

extract of alligator pepper (Zingiberaceae aframomum melegueta) on gestational weight gain. Niger J Physiol Sci. 2009 Dec;24(2):165–9.

REGULATORY BACKGROUND

Aframomum melegueta is listed in (see below):

- The Old Dietary Ingredient list, 2011, UNPA
- The Herbs of Commerce, First edition, 1992, AHPA
- The American Herbal Products Association's Botanical Safety Handbook 1997

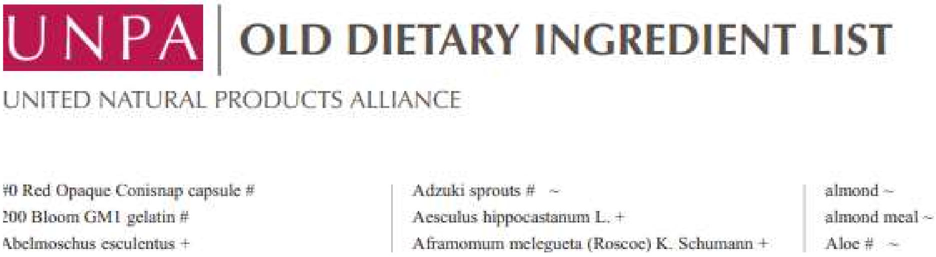

UNPA | OLD DIETARY INGREDIENT LIST
UNITED NATURAL PRODUCTS ALLIANCE

#0 Red Opaque Conisnap capsule #
200 Bloom GM1 gelatin #
Abelmoschus esculentus +
Adzuki sprouts # ~
Aesculus hippocastanum L. +
Aframomum melegueta (Roscoe) K. Schumann +
almond ~
almond meal ~
Aloe # ~

Figura 1: Image from "Old Dietary Ingredient List (UNPA)".

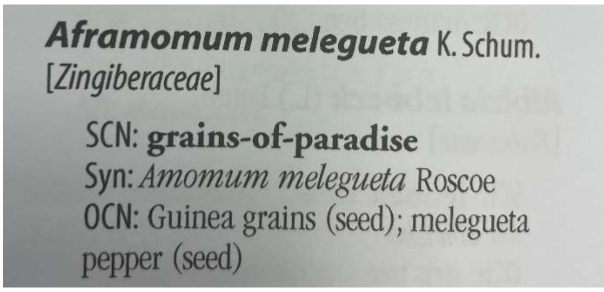

Aframomum melegueta K. Schum.
[Zingiberaceae]
SCN: **grains-of-paradise**
Syn: *Amomum melegueta* Roscoe
OCN: Guinea grains (seed); melegueta pepper (seed)

Figura 2: Image from "Herbs of commerce".

Moreover, Aframomum melegueta Rosc. ("Grains of paradise") synonym of Aframomum melegueta K. schum, is mentioned in the Code of Federal Regulations Title 21 "21 CFR – PART 182 – SUBSTANCES GENERALLY RECOGNIZED AS SAFE – Sec. 182.10 Spices and other natural seasonings and flavorings". Aframomum melegueta is used as spice and is generally recognized as safe for its intended use.

21 CFR Part 182 (up to date as of 10/06/2022)
Substances Generally Recognized as Safe

Common name	Botanical name of plant source
Camomile (chamomile), German or Hungarian	Matricaria chamomilla L.
Capers	Capparis spinosa L.
Capsicum	Capsicum frutescens L. or Capsicum annuum L.
Caraway	Carum carvi L.
Caraway, black (black cumin)	Nigella sativa L.
Cardamom (cardamon)	Elettaria cardamomum Maton.
Cassia, Chinese	Cinnamomum cassia Blume.
Cassia, Padang or Batavia	Cinnamomum burmanni Blume.
Cassia, Saigon	Cinnamomum loureirii Nees.
Cayenne pepper	Capsicum frutescens L. or Capsicum annuum L.
Celery seed	Apium graveolens L.
Chervil	Anthriscus cerefolium (L.) Hoffm.
Chives	Allium schoenoprasum L.
Cinnamon, Ceylon	Cinnamomum zeylanicum Nees.
Cinnamon, Chinese	Cinnamomum cassia Blume.
Cinnamon, Saigon	Cinnamomum loureirii Nees.
Clary (clary sage)	Salvia sclarea L.
Clover	Trifolium spp.
Coriander	Coriandrum sativum L.
Cumin (cummin)	Cuminum cyminum L.
Cumin, black (black caraway)	Nigella sativa L.
Elder flowers	Sambucus canadensis L.
Fennel, common	Foeniculum vulgare Mill.
Fennel, sweet (finocchio, Florence fennel)	Foeniculum vulgare Mill. var. dulce (DC.) Alex.
Fenugreek	Trigonella foenum-graecum L.
Galanga (galangal)	Alpinia officinarum Hance.
Geranium	Pelargonium spp.
Ginger	Zingiber officinale Rosc.
Grains of paradise	Amomum melegueta Rosc.

Image 3: Image from "Code of Feneral Regulations".

nektium

SPECIFICATION SHEET

Product name: *Aframomum melegueta* extract 7-10% total gingerols
Common name: **Alligator pepper**
Code: AME07S

01 Product description	Specification	Test method
Plant part used	Seeds	Visual
Botanical name	*Aframomum melegueta*	Macroscopic
Carrier(s) used	Arabic gum: citrus pectin: pea protein (2:1:1; w:w:w)	≥ 67%
Plant: Extract ratio	5-8:1	By weight

02 Physical data	Specification	Test method
Appearance	Free flowing powder	Visual
Color	Light brown	Visual
Aroma	Characteristic	Organoleptic
Flavor	Spicy, characteristic	Organoleptic
Particle Size	100 % Through 80 Mesh	US standard sieve
Solubility in Water	Practically insoluble	EU Pharm. 5.11.
Bulk Density	0.3-0.5 (g/cm^3)	USP 616 / EU Pharm. 2.9.34.

03 Chemical data	Specification	Test method
Total gingerols (capsaicin eq.)	7.0% - 10.0 %	UPLC (MUPLCXX)
Moisture	≤ 7.0 %	USP 921
Total heavy metal	TBD	EU Pharmacopeia 2.4.8.
Total arsenic	TBD	ICP-MS
Lead	TBD	ICP-MS
Cadmium	TBD	ICP-MS
Mercury	TBD	ICP-MS
Residual solvents	TBD	GC-MS (USP 467 / Directive (EU)2009/32 and 2016/1855)
Pesticides	Complies with USP 565 and Regulation 396/2005/CE and amendments	
Contaminants	Complies with Regulation EC 1881/2006 and amendments	

04 Microbiological data	Specification	Test method
Total plate count	TBD	ISO 4833
Yeast & mold	TBD	ISO 7954
Salmonella	TBD	ISO 6579
E. coli	TBD	EU Pharm. 2.6.13.
Total Coliforms	TBD	M0042 - Part V. VRB Plate Count
Staphylococcus aureus	TBD	EU Pharm. 2.6.13.

05 Additional information

Extraction method	Water: ethanol (30:70) extraction and spray dried
Packing	25 kg HDPE drums with double PE bags
Storage	Original container in a cool, dry place
Shelf life	TBD years (currently under evaluation)
Country of origin /manufactured	Spain
Intended use	Nutraceutical
Non-Allergens/Gluten	This product does not contain any of the food allergens cited in the Regulation (EU) No 1169/2011
Non-BSE/TSE	All ingredients in the product are of vegetable origin
Non-Irradiation	This material has not been subjected to irradiation
Nanomaterial	This material is not produced using Nanotechnology and is free from Nanoparticles
Non-GMO status	This product is neither considered genetically modified nor derived from any genetically modified organisms, as defined by the EC regulations 1830/2003/EC, 1829/2003/EC and any amending legislation

Ver. draft
31/01/2022

Company Certification

GMP Certified by MERIEUX NutriScience

Process Technology Manager
Tanausú Vega

nektium

DRAFT

HAMILTON ANXIETY RATING SCALE (HAM-A)

Reference: Hamilton M. (1959). The assessment of anxiety states by rating. Br J Med Pyschol, 32, 50-55.

The following is a series of phrases that refer to the reactions people commonly display when faced with life conflicting situations.

Read the questionnaire carefully and try to identify with an X the reactions which have that have appeared in you:

0) Never
1) Sometimes
2) Several times
3) Almost always
4) Always

QUESTIONNAIRE		ANSWERS				
1	I have a feeling that something bad might happen to me, that the worst might happen to me. I feel worried, irritable.	0	1	2	3	4
2	I have the feeling of restlessness and I can't relax. I get tired easily. I startle. I tremble. I cry easily					
3	I'm afraid of the dark, of being alone, of unknown people, of animals, of traffic, of crowds.					
4	I have difficulty falling asleep. I wake up several times. I wake up tired. No restful sleep.					
5	I have a bad memory, especially of recent events. I I find it hard to concentrate.					
6	I have no interest in my surroundings. I feel sad. I I feel sleepy during the day and I can't fall asleep at night.					
7	I feel muscle aches and pains. My teeth grind. I have a weak and insecure voice.					
8	I have ringing in my ears. Blurred vision. I feel waves of heat or cold. I have itching and a feeling of weakness.					
9	I feel my heart beating faster than usual. I feel palpitations, chest pains, agitation.					
10	I have a feeling of suffocation and shortness of breath. I need to breathe. I feel a tightness or pain in my chest.					
11	I have difficulty swallowing. I feel burning, heaviness or gastric fullness. gastric fullness. I feel nausea and vomiting. Constipation.					
12	I feel the need to urinate frequently, even if only a little. I have hormonal disturbances. My sex life has deteriorated					
13	My mouth feels dry. I blush and suffer easily. I get dizzy and I get dizzy and things spin around. My hair stands on end.					
14	I have felt uncomfortable, uneasy, tense, impatient, my hands are sweating and my pulse is racing as I answer these questions.					

PROTOCOL FOR THE APPLICATION OF THE POMS TEST

NAME: ______________________________ DATE: _____________

FOR EACH ASPECT, PLEASE INDICATE THE ANSWER YOU CONSIDER ACCORDING TO THE SCALE. REMEMBER TO CHECK THAT EACH ASPECT IS ANSWERED, MAKE SURE THAT YOU ONLY ANSWER WITH ONE INDICATOR.

Carefully read the list of words described in the quiz below, these words describe feelings that people have. After reading each word, look at the five options above and choose the one that best describes how you have felt during the last 24 hours. Select a value in the box to the right of each word. Do not leave any boxes blank.

- QUESTIONS

1. Tense	20. Fatigued
2. Unstable	21. Exhausted
3. Nerves on edge	22. Lazy
4. Frightened	23. Dejected
5. Relaxed	24. Lazy
6. Restless	25. Unhappy
7. Uneasy	26. Regretful for things done
8. Nervous	27. Sad
9. Anxious	28. Melancholic
10. Lively	29. Hopeless
11. Active	30. Unworthy
12. Energetic	31. Discouraged
13. In a Good mood	32. Alone
14. Alert	33. Miserable
15. Full of dynamism	34. Pessimistic
16. Carefree	35. Despairing
17. Vigorous	36. Useless
18. worn-out	37. Terrified
19. Apathetic	38. Guilty

NOTHING				LITTLE			MODERATE				QUITE			A LOT			
0				1			2				3			4			
	0	1	2	3	4		0	1	2	3	4		0	1	2	3	4
1. Tense						17. Vigorous						33. Miserable					
2. Unstable						18. Worn-out						34. Pessimistic					
3. Nerves on Edge						19. Apathetic						35. Despairing					
4. Frightened						20. Fatigued						36. Useless					
5. Relaxed						21. Exhausted						37.Terrified					
6. Restless						22. Lazy						38. Guilty					
7. Uneasy						23. Dejected											
8. Nervous						24. Very tired											
9. Anxious						25. Unhappy											
10. Lively						26. Regretful for things done											
11. Active						27. Sad											
12. Energetic						28. Melancholic											
13. Good Mood						29. Hopeless											
14. Alert						30. Unworthy											
15. Full dynamism						31. Discouraged											
16. Carefree						32. Alone											

Pittsburgh Sleep Quality Index (PSQI)

Name: Date:

1. During the last month, what was your usual bedtime?

2. ¿ How long did it normally take you to fall asleep during the last month?
    1. Less than 15 min.
    2. Between 16-30 min.
    3. Between 31-60 min.
    4. More than 60 min.

3. During the last month, what time did you usually get up in the morning?

4. How many hours do you estimate you have actually slept each night in the last month?

5. During the last month, how many times have you had problems sleeping because of:<br>a) Not being able to fall asleep in the first half hour:	1. Not at all in the last month<br>2. Less than once a week<br>3.Once or twice a week<br>4.Three or more times a week
b) Waking up during the night or in the early hours of the morning	1. Not at all in the last month<br>2. Less than once a week<br>3.Once or twice a week<br>4.Three or more times a week
c) Having to get up to go to the toilet	1. Not at all in the last month<br>2. Less than once a week<br>3.Once or twice a week<br>4.Three or more times a week
d) Not being able to breathe properly	1. Not at all in the last month<br>2. Less than once a week<br>3.Once or twice a week<br>4.Three or more times a week
e) Coughing or snoring noisily	1. Not at all in the last month<br>2. Less than once a week<br>3.Once or twice a week<br>4.Three or more times a week

f) Feeling cold	1. Not at all in the last month<br>2. Less than once a week<br>3.Once or twice a week<br>4.Three or more times a week
g) Feeling too hot	1. Not at all in the last month<br>2. Less than once a week<br>3.Once or twice a week<br>4.Three or more times a week
h) Having nightmares or bad dreams	1. Not at all in the last month<br>2. Less than once a week<br>3.Once or twice a week<br>4.Three or more times a week
h) suffer pain	1. Not at all in the last month<br>2. Less than once a week<br>3.Once or twice a week<br>4.Three or more times a week
i) Other reasons	1. Not at all in the last month<br>2. Less than once a week<br>3.Once or twice a week<br>4.Three or more times a week
6. Over the last month, how would you rate the quality of your sleep overall?	1. Very good<br>2. Quite good<br>3. Quite bad<br>4. Very bad
7. During the last month, how many times have you taken medicine (on your own or prescribed by your doctor) to sleep?	1. Not at all in the last month<br>2. Less than once a week<br>3.Once or twice a week<br>4.Three or more times a week
8. During the last month, how many times have you felt drowsy while driving, eating or doing any other activity?	1. Not at all in the last month<br>2. Less than once a week<br>3.Once or twice a week<br>4.Three or more times a week
9. During the last month, have you had much trouble getting into the mood to do any of the activities listed in the previous question?	1. No problem<br>2. Just a slight problem<br>3. One problem<br>4. A serious problem

10. Do you sleep alone or in company?	1. Alone<br>2. With someone in another room<br>3. Same room, but a different bed<br>4. In the same bed

LEEDS SLEEP EVALUATION QUESTIONNAIRE

Name:

1. BAR 2. REGULAR 3. GOOD 4. EXCELENT

1. How would you describe the way you sleep nowadays compared to what you usually do?

2. How would you describe the quality of your sleep compared to normal sleep?

3. How would you describe your awakening in comparison to the usual?

4. How did you feel when you woke up?

5. How do you feel now?

6. How would you describe your balance and coordination when you stand up?

ANSWERS

Questions		1	2	3	4	5	6
Date							
Date							
Date							

Daily information

DAY1

QUESTION	YES	NOT	OBSERVATIONS
Have you carried out routine daily activities (work, shopping, etc.) today?			
Have you done anything different (excursion, dinner with friends, etc.) today?			
Have you done any physical activity (gym, running, etc.) today?			
Have you eaten between 3 and 5 meals today?			
Have you eaten at least 3 pieces of fruit or vegetables today?			
Have you had a headache?			
Have you had any digestive problems (tummy ache, constipation, etc.)?			
Have you felt more fatigued or tired than on other days?			

DAY 2

QUESTION	YES	NOT	OBSERVATION
Have you carried out routine daily activities (work, shopping, etc.) today?			
Have you done anything different (excursion, dinner with friends, etc.) today?			
Have you done any physical activity (gym, running, etc.) today?			
Have you eaten between 3 and 5 meals today?			
Have you eaten at least 3 pieces of fruit or vegetables today?			
Have you had a headache?			
Have you had any digestive problems (tummy ache, constipation, etc.)?			
Have you felt more fatigued or tired than on other days?			

Universitat d'Alacant
Universidad de Alicante

Parque Científico Alicante

KINETIC PERFORMANCE

Research Ethics Committee

Alicante, Thursday 12 January 2023

Gathered together at the Alicante Science Park, the Research Ethics Committee of Kinetic Performance, at its meeting of 09-01-2023 (minutes 02/23), evaluated the research project:

TITLE: Effects of a vegetable extract modulator of the endocannabinoid system and its anxiolytic capacity on senior participants in situations of stress or anxiety.

Registration No.: K04/023

Promoter code: AME_HCT_2023

Applicant researcher: Carlos Elvira Aranda

Decision: APPROVED (09-01-23)

This Research Ethics Committee, in accordance with Article 5, considers that the aforementioned project, being the promoter Nektium, is **ethically and methodologically acceptable. It thus complies with international GCP standards (CPMP/ICH/135/95)**. In addition, it also considers that the investigators who requested the evaluation of this Committee are competent to carry out this project, which is framed within the priority research lines of the Alicante Science Park and Kinetic Performance.

DEPARTAMENTO DE DIDÁCTICA GENERAL
Y DIDÁCTICAS ESPECÍFICAS
UNIVERSIDAD DE ALICANTE

Sgd.: Dr. José Antonio Pérez Turpin

R.E.C. President

INFORM CONSENT

Mr./Ms.____

with DNI____.

I MANIFEST THAT:

1. I have received from the company__________________________. all necessary information in a confidential, clear, understandable and satisfactory manner about the nature and purpose of the objectives, procedures and timing to be followed in the course of the scientific study on____ managed by the company____.
2. I agree to the processing of my personal data, including medical, anthropometric and other data, for the purposes of the study as described in the Data Protection Act General Data Protection Regulation (Regulation EU 2016-679 of the European Parliament and of the Council of 27 April 2016) and the Spanish regulations on personal data protection in force.
3. I also consent to the release of study-related data, such as my age, gender, or personal medical information, to the study sponsor.
4. I consent to the storage and use of the biological samples (blood samples) collected from me for the purposes of this study.
5. I agree that, once I have received all the necessary information, I agree and undertake to participate in this scientific study.
6. I agree to participate in this study on a voluntary basis and understand that I may withdraw from this study at any time without cause.

Sign of Participant Date

Name and Surname of the participant

I have informed the patient about this study and answered all the questions raised

Signature of the person giving informed consent Date

Name and Surname of the person providing informed consent
