# Supplementary material for: Aframomum melegueta Seed Extract’s Effects on Anxiety, Stress, Mood, and Sleep: A Randomized, Double-Blind, Pilot Clinical Trial
Source: Pharmaceuticals (Basel). 2025 Feb 19;18(2):278. doi: 10.3390/ph18020278 (PMC11859572; doi:10.3390/ph18020278)
Supplement: Supplementary file 1 [file pharmaceuticals-18-00278-s001.zip › S5 File. Study protocol Spanish Committe (in Spanish).pdf]

Efectos de un extracto vegetal modulador del sistema endocanabinoide y su capacidad ansiolítica sobre personas senior en situaciones de estrés o ansiedad.

PROTOCOLO

PROMOTOR DEL ESTUDIO

Nektium Pharma S.L.

C/ Las Mimosas 8, Polígono Industrial de Arinaga, 35118, Agüimes, las Palmas

EMPRESA/GRUPO INVESTIGACIÓN DEL ESTUDIO

Kinetic Performance S.L.

Nuevos Institutos del Parque Científico de la Universidad de Alicante, en Carretera de San Vicente s/n 03690, San Vicente del Raspeig (Alicante).

INVESTIGADOR PRINCIPAL DEL ESTUDIO

Dr. D. Carlos Elvira Aranda

Jefe Investigación Kinetic Performance S.L.

Nuevos Institutos del Parque Científico de la Universidad de Alicante, en Carretera de San Vicente s/n 03690, San Vicente del Raspeig (Alicante).

ÍNDICE

1. Información general……………………………………………………………………..4
2. Objetivos………………………………………………………………………………..6
3. Diseño…………………………………………………………………………………..6
   3.1. Descripción del diseño del estudio……………………………………………6
4. Selección de participantes……………………………………………………………7
   4.1. Criterios inclusión…………………………………………………………………7
   4.2. Criterios de exclusión……………………………………………………………..7
   4.3. Criterios de retirada……………………………………………………………….7
5. Tratamiento en la investigación……………………………………………………..8
   5.1. Descripción del tratamiento………………………………………………………8
   5.2. Medicación permitida y prohibida………………………………………………..8
   5.3. Monitorización del cumplimiento…………………………………………………8
6. Procedimiento y variables……………………………………………………………..9
7. Calendario y visitas del estudio……………………………………………………..11
8. Acontecimientos adversos……………………………………………………………14
   8.1. Definiciones………………………………………………………………………..14
   8.2. Procedimiento………………………………………………………………………15
   8.3. Notificación………………………………………………………………………….16
   8.4. Información a los investigadores…………………………………………………16
9. Aspectos éticos…………………………………………………………………………17
   9.1. Consideraciones generales……………………………………………………….17
   9.2. Información al paciente……………………………………………………………17
   9.3. Confidencialidad y acceso a los datos…………………………………………..19
10. Consideraciones prácticas……………………………………………………………19
   10.1. Responsabilidades de todos los participante en el ensayo…………………19
   10.2. Desviaciones del protocolo………………………………………………………20
   10.3. Enmiendas al protocolo…………………………………………………………..20
   10.4. Aceptación del investigador………………………………………………………20
   10.5. Compromiso de publicación de resultados…………………………………….20
11. Referencias………………………………………………………………………………21
12. Anexos……………………………………………………………………………………21

1. INFORMACIÓN GENERAL

Investigación sobre los efectos de un extracto vegetal modulador del sistema endocanabinoide y su capacidad ansiolítica sobre adultos mayores.

Grado evidencia	Fecha	Número registro CEI	Código estudio
Estudio clínico aleatorizado	07/12/2022	K04/023	AME_HCT_2023

PROMOTOR

Nektium Pharma S.L. (C/ Las Mimosas 8, Polígono Industrial de Arinaga, 35118, Agüimes, las Palmas)

TÍTULO DEL ESTUDIO

Efectos de un extracto vegetal modulador del sistema endocanabinoide y su capacidad ansiolítica sobre personas senior en situaciones de estrés o ansiedad.

CÓDIGO DEL PROTOCOLO

AME_HCT_2023

EQUIPO INVESTIGADOR

- Carlos Elvira Aranda (IP). *Kinetic Performance S.L. Universidad de Alicante. GICAFD.*
- Mario Terol Sanchis (Coord.). *Kinetic Performance S.L. Universidad de Alicante. GICAFD. Centro de Ejercicio Terapéutico GanaSalud.*
- Óscar Feltrer Hernández. *Kinetic Performance S.L. Universidad de Alicante. GICAFD.*
- Loreto Lledó Rico. *Kinetic Performance S.L.*

- Jaime Enrique Gómez Paternina. *Kinetic Performance S.L.*
- Francisco Bailén Gómez. *Centro de Ejercicio Terapéutico GanaSalud.*
- Rubén Acame Rocamora. *Centro de Ejercicio Terapéutico GanaSalud.*
- Sergio Pérez Galiana. *Kinetic Performance S.L. Universidad de Alicante. GICAFD.*
- Héctor Manuel Bernabé Lorca. *Kinetic Performance S.L. Universidad de Alicante. GICAFD.*
- José Palomares Baeza. *Centro Inmunológico de Alicante (CIALAB) - Laboratorio Clínico de Ribera Salud (Riberalab).*

CENTROS DE REALIZACIÓN DEL ESTUDIO

- Kinetic Performance S.L.
- Parque Científico de Alicante
- Universidad de Alicante
- Centro de Ejercicio Terapéutico GanaSalud
- Centro Inmunológico de Alicante (CIALAB) asociado a Ribera Salud como Laboratorio Clínico (Riberalab).

COMITÉ ÉTICO DE INVESTIGACIÓN

Comité Ético de Investigación perteneciente a Kinetic Performance (Parque Científico Alicante).

2. OBJETIVOS

Objetivo principal

Evaluar el efecto ansiolítico de un extracto vegetal modulador del sistema endocanabinoide en personas mayores que experimentan situaciones de ansiedad.

Objetivos específicos

- Determinar el efecto del producto a distintas dosis y su respuesta sobre la dimensión ansiedad.
- Analizar los cambios sobre el mood (estado de ánimo) provocados por el extracto vegetal.
- Determinar la influencia del extracto vegetal sobre parámetros cualitativos del sueño nocturno.
- Evaluar la sensibilidad de los parámetros de variabilidad de la frecuencia cardíaca durante el sueño nocturno.
- Evaluar la respuesta fisiológica a través de indicares inflamatorios durante a las distintas dosis administradas.

3. DISEÑO DEL ESTUDIO

3.1. Descripción del diseño del estudio

El estudio se realizará utilizando un diseño experimental cruzado de comparación con un grupo aleatorizado y doble ciego. Se utilizará un único grupo que recibirá cuatro dosis del producto: 0mg, 50mg, 100mg y 150mg durante un período de tres días. Entre cada intervención se cumplirá un periodo de lavado de siete días.

La aplicación de la dosis será aleatorizada bajo códigos de randomización y seguirá dos perfiles distintos:

- **Ramp up:** 0mg - 50mg - 100mg - 150mg
- **Ramp down:** 150mg - 100mg - 50mg - 0mg

4. PARTICIPANTES

El grupo de participantes estará compuesto por 37 personas mayores (de 40 a 50 años) que experimenten un estado de ansiedad y que serán asignados aleatoriamente para recibir la dosis de tratamiento.

4.1. Criterios de inclusión

1. Edad entre 40 y 50 años.
2. Puntuación superior a 18 puntos en la Escala de Ansiedad de Hamilton (HAM-A).
3. Aceptación y firma del consentimiento informado para el estudio tras haber recibido la información adecuada.

4.2. Criterios de exclusión

1. Puntuación superior a 20 puntos en la Escala de Depresión de Hamilton (HDRS).
2. Recepción de tratamiento médico para la ansiedad, el estrés o la depresión.
3. Dependencia de drogas y alcohol.
4. Trastornos de personalidad graves que puedan interferir en la participación en el estudio (psicosis, ideación suicida intensa, etc.).
5. En el caso de mujeres, tener intención de quedarse embarazada.
6. Trastornos epilépticos.
7. Trastornos hepáticos (cirrosis, hepatitis, etcétera).
8. Deportistas profesionales o que frecuenten actividades físicas extremas.
9. Imposibilidad de completar el periodo de intervención por factores externos.

4.3. Criterios de retirada.

Todos los participantes podrá ser retirado si cumple en cualquier momento del estudio alguno de los siguientes criterios de retirada:

1. Incumplimiento de alguno de los otros criterios de inclusión durante el estudio.
2. Cumplimiento de alguno de los criterios de exclusión durante el estudio.
3. Toxicidad relacionada con el producto.
4. Incumplimiento de la obtención de las variables obligatorias.
5. Incumplimiento de los plazos establecidos.
6. Pérdida de seguimiento.
7. Fallecimiento.

5. TRATAMIENTO EN LA INVESTIGACIÓN

5.1. Descripción del tratamiento

Todos los participantes recibirán cuatro dosis del producto: 0mg, 50mg, 100mg, 150mg. El protocolo de toma y período de lavado se detalla en el apartado de diseño del protocolo. La información del producto se detalla en ANEXO 1.

5.2. Medicación permitida y prohibida.

Se permite toda medicación que no influya en los siguientes aspectos:

- Medicación recetada para el tratamiento de la ansiedad, estrés o depresión, o cualquier tratamiento que pueda afectar a estos trastornos.
- Alteración en parámetros del sueño.
- Alteración en los sistemas parasimpático y simpático.
- Queda prohibido toda droga o ingesta excesiva de alcohol.

5.3. Monitorización del cumplimiento

El cumplimiento de la tratamiento por parte del participante se evaluará en cada visita por el personal investigador.

6. PROCEDIMIENTO Y VARIABLES

El reclutamiento de candidatos será realizado por el equipo investigador en conjunto con los centros colaboradores. Los centros participantes en este estudio son la empresa de base tecnológica Kinetic Performance S.L., la Universidad de Alicante, el Parque Científico de Alicante y el Centro de Ejercicio Terapéutico GanaSalud. No se ha establecido ningún material específico para el reclutamiento de participante, como carteles o anuncios. Los participantes serán seleccionados entre los profesionales que trabajen en cada centro y que sean candidatos a la inclusión en base con los criterios de inclusión y exclusión definidos. Todos los participantes seleccionados para su posible inclusión deberán ser citados para una primera visita de selección presencial con el médico participante y el investigador principal o coordinador del estudio, donde se evaluará su adecuación al estudio en función de los criterios de inclusión y exclusión, les informará del estudio y se recabará por escrito su consentimiento para la participación.

Se considerará como primer acto de selección la fecha de firma del consentimiento informado por el primer participante.

El protocolo del estudio incluye varios procedimientos y variables para evaluar el efecto del extracto vegetal en los participantes. Estos son los procedimientos y variables mencionados:

- **Variables antropométricas:** Día 0 (inicio tratamiento) y +2 (fin de tratamiento). Medición de composición corporal: Se realizarán mediciones de la edad, altura y peso de los participantes. Además, se evaluará la composición corporal, incluyendo la masa grasa, el porcentaje de masa grasa, la masa muscular, el porcentaje de masa muscular y el índice de masa corporal (IMC). Estos datos ayudarán a caracterizar a los participantes y a tener en cuenta posibles factores de confusión relacionados con la composición corporal.
- **Tensión arterial:** Día 0 (inicio tratamiento) y +2 (fin de tratamiento). Se medirá la presión arterial de los participantes utilizando un dispositivo de monitorización

automática de la presión arterial. Esta medición es importante para evaluar la función cardiovascular de los participantes y detectar posibles cambios relacionados con la intervención del extracto vegetal.

- **Análisis bioquímico:** Día 0 (inicio tratamiento) y +2 (fin de tratamiento). Se llevarán a cabo pruebas de laboratorio para medir diversos parámetros bioquímicos en muestras de sangre de los participantes. Estos análisis incluirán el hemograma completo para evaluar la composición de las células sanguíneas. También se medirán los niveles de sodio, cloro, magnesio y zinc, que son minerales relevantes para el funcionamiento metabólico y celular. Asimismo, se analizarán las enzimas GGT, GPT, GOT y FA, que son indicadores de la función hepática. Se medirá la proteína C reactiva, que es un marcador de la inflamación sistémica, y se evaluarán las citocinas proinflamatorias IL-1, IL-6, IL-8 y TNF-α. Por último, se medirá el cortisol basal en suero, que es una hormona relacionada con la respuesta al estrés.
- **Escala de Ansiedad de Hamilton (HAM-A):** Día 0 (inicio tratamiento) y +2 (fin de tratamiento). ANEXO 2.
- **Test Profile of Mood States (POMS):** Día 0 (inicio tratamiento) y +2 (fin de tratamiento). ANEXO 3.
- **Índice de Calidad de Sueño de Pittsburgh (PSQI):** Día 0 (inicio tratamiento) y +2 (fin de tratamiento). ANEXO 4.
- **Cuestionario de Evaluación del Sueño de Leeds (LSEQ):** Día 0 (inicio tratamiento), +1 y +2 (fin de tratamiento). ANEXO 5.
- **Variabilidad de la frecuencia cardíaca (VFC):** Día 0 (inicio tratamiento), +1 y +2 (fin de tratamiento). La variabilidad de la frecuencia cardíaca se recogerá con bandas de frecuencia cardíaca POLAR H10+ durante las horas de sueño.
- **Diario del paciente:** Día 0 (inicio tratamiento), +1 y +2 (fin de tratamiento). Se proporcionará a los participantes un cuaderno en el que registrarán diariamente

información relevante sobre sus actividades, la ingesta de alimentos, la presencia de síntomas como dolor de cabeza o problemas digestivos, así como la fatiga y cualquier otro aspecto que pueda ser relevante para el estudio (ANEXO 6). Estos registros permitirán obtener información subjetiva y detallada sobre el día a día de los participantes durante el estudio.

Durante todo el periodo de intervención (detallada su duración en el apartado 7), los participantes seguirán un programa de entrenamiento normalizado, basado en intensidades, RPE o RIR:

- **Volumen semanal:** 2-3 sesiones x semana (1:1 - Fuerza-Resistencia ; 2:1 - Fuerza-Resistencia).
- **Volumen sesión:** 1 hora (NO INFERIOR A 45 MINUTOS).
- **Intensidad**: 70-85% / RPE 6-8 / RIR 2-3
- **Densidad**: 1:2
- **Ent. Fuerza**: Priorizan ejercicios multiarticulares. FullBody (No cluster. No Weider).
- **Ent. Cardiovascular:** Intervalos absolutos entre 10-15’ para RPE 7-8. Intervalos relativos entre 30”-1’. Priorizan por orden bicicleta, elíptica, cinta.

7. CALENDARIO Y VISITAS DEL ESTUDIO

La duración total del estudio se estima en 18 semanas desde el inicio del reclutamiento hasta la última visita del participante:

- Período de reclutamiento: 8 semanas
- Periodo de tratamiento: 10 semanas
- Periodo individual del estudio: 33 días.

Las visitas de cada paciente en el estudio se realizan en horario de mañana, cumpliendo la ventana temporal de 7.00 a 10.30h. Los participantes deben asistir en estado de ayuno, evitando sustancias estimulantes (ej. café). El procedimiento de visitas se detalla a continuación:

- **Día 0. Inicio tratamiento (Visita):**
    - Anamnesis.
    - Tensión arterial.
    - Variables antropométricas
    - Escala de Ansiedad de Hamilton (HAM-A)
    - Test Profile of Mood States (POMS).
    - Índice de Calidad de Sueño de Pittsburgh (PSQI).
    - Cuestionario de Evaluación del Sueño de Leeds (LSEQ).
    - Extracción sanguínea (variables bioquímicas).
    - Variabilidad de la frecuencia cardíaca* (durante el sueño nocturno).
    - Diario del paciente*.

- **Día +1. intervención (No visita):**
    - Cuestionario de Evaluación del Sueño de Leeds (LSEQ).
    - Variabilidad de la frecuencia cardíaca (durante el sueño nocturno).
    - Diario del paciente.

- **Día +2. Fin de tratamiento (Visita):**
    - Anamnesis.
    - Tensión arterial.
    - Variables antropométricas

- Escala de Ansiedad de Hamilton (HAM-A)
- Test Profile of Mood States (POMS).
- Índice de Calidad de Sueño de Pittsburgh (PSQI).
- Cuestionario de Evaluación del Sueño de Leeds (LSEQ).
- Extracción sanguínea (variables bioquímicas).
- Variabilidad de la frecuencia cardíaca* (durante el sueño nocturno).
- Diario del paciente*.

- **Día +3 a +9. Periodo de lavado (No visita):**
  - Descanso del participante. Custodia de la banda de frecuencia cardíaca o devolución en estos días si finaliza estudio.

*Las variables no se realizan en presencia del investigador.

Tabla 1.

Descripción del período de intervención.

	DÍA 0	DÍA +1	DÍA +2
	**Inicio**	**Tratamiento**	**Fin**
**Anamnesis**			
**Constantes vitales[1]**			
**Mediciones antropométricas**			
**Analítica**			
**HAM-A**			
**POMS**			
**PSQI**			
**LSEQ**			
**VFC**			
**Diario del paciente**			
**Acontecimientos adversos**			

(1) Tensión arterial

8. ACONTECIMIENTOS ADVERSOS

8.1. Definiciones

Acontecimiento Adverso (AA)

Cualquier incidencia perjudicial para la salud en un paciente o sujeto de ensayo clínico tratado con un medicamento, aunque no tenga necesariamente relación causal con dicho tratamiento.

Reacción Adversa (RA)

Una RA es toda reacción nociva y no intencionada a un medicamento en investigación, independientemente de la dosis administrada.

Acontecimiento Adverso Grave (AAG) y Reacción Adversa Grave (RAG)

Se consideran graves los AA o RA que, a cualquier dosis puedan provocar la muerte, amenazar la vida del sujeto, requerir la hospitalización del paciente o prolongar una hospitalización existente, provocar invalidez o incapacidad permanente o importante, o dar lugar a una anomalía o malformación congénita. También se consideran graves aquellas sospechas de AA o RA importantes desde el punto de vista médico, aunque no cumplan los criterios anteriores, incluyendo los acontecimientos médicos importantes que requieran una intervención para evitar que se produzca una de las consecuencias anteriormente descritas. Asimismo, se notificarán como graves todas las sospechas de transmisión de un agente infeccioso a través de un medicamento. El concepto "amenazar la vida del sujeto" en la definición se refiere a que en opinión del investigador, el paciente en el momento del AA o RA está en riesgo real de muerte; no se refiere a que el AA/RA hipotéticamente pudiera haber ocasionado la muerte en caso de haber sido más intenso.

Reacción Adversa Inesperada (RAI)

Cualquier RA cuya naturaleza, intensidad o consecuencias no se corresponde con la información de seguridad de referencia.

Reacción Adversa Grave e Inesperada (RAGI)

RAG (definida previamente), cuya naturaleza, gravedad o consecuencias no se corresponde con la información de seguridad de referencia.

8.2. Procedimiento

El investigador recogerá sistemáticamente y hará el seguimiento de los AA desde la primera administración del producto en investigación hasta la visita final de seguimiento de cada participante. Todos los AA referidos espontáneamente por el participante y/o en respuesta a una pregunta abierta por parte del investigador, o aquellos observados u objetivados en la

exploración física o en alguna prueba complementaria se registrarán en el CRD del participante.

Todos los AAG experimentados por un participante, independientemente de su presunta causalidad, se monitorizarán hasta que el acontecimiento se haya resuelto o estabilizado, los valores anormales de laboratorio que hayan dado lugar a la Reacción Adversa o al AA grave hayan regresado a los valores basales o se hayan estabilizado a un nivel aceptable para el investigador, haya una explicación satisfactoria para los cambios observados o no se haya podido llevar a cabo el seguimiento del paciente. Las Reacciones Adversas se monitorizarán hasta su resolución o estabilización (lo que ocurra primero).

El investigador evaluará y registrará con detalle los AA, incluyendo la fecha de inicio y final, la descripción del acontecimiento, la gravedad, la evolución, el desenlace, la relación del AA con el medicamento de investigación y las medidas adoptadas (tratamientos, exploraciones complementarias adicionales).

La decisión sobre si la intensidad de un AA es suficiente para retirar al paciente del estudio será tomada según el criterio clínico del investigador. El paciente también puede decidir retirarse del estudio si según su criterio si considera que la intensidad del AA no es soportable.

8.3. Notificación

En caso de que se produzca un AAG, el investigador lo comunicará al promotor o a quien asuma las tareas delegadas por el promotor en un plazo máximo de 24 horas a partir del momento en que tenga conocimiento de él.

8.4. Información a los investigadores

El investigador principal (IP) se encargará de gestionar la información relativa a este aspecto entre el promotor y el equipo investigador.

9. ASPECTOS ÉTICOS

9.1. Consideraciones generales

Este estudio deberá desarrollarse de acuerdo con el protocolo y con la normas de buena práctica clínica, tal como se describe en la legislación vigente aplicable. El equipo investigador accede a seguir las instrucciones y procedimientos descritos en el protocolo y por lo tanto cumplirá los principios de Buena Práctica Clínica en los cuales se basa. Las modificaciones en el protocolo serán acordadas tanto por el promotor como el investigador principal, y con el consentimiento del Comité Ético de Investigación (CEI).

9.2. Información al paciente

Durante la visita de selección, el investigador será responsable de informar de forma completa y en profundidad al participante de todos los aspectos pertinentes del estudio, naturaleza y objetivos del estudio, así como los posibles riesgos que conlleva, incluyendo la información escrita y el dictamen favorable del comité ético (ANEXO 7). El lenguaje utilizado en la información oral no deberá ser técnico, sino práctico y deberá poder ser entendido por el participante. Además, la información no debe contener ningún tipo de lenguaje que lleve a renunciar o a parecer que renuncia a cualquier derecho legal, o que libere o parezca que libera al investigador, al centro, al promotor o a su personal de sus obligaciones en caso de negligencia. Ni el investigador, ni el personal del estudio deberán coaccionar o influir indebidamente al sujeto para que participe en el estudio. De forma específica, se informará al sujeto de los siguientes detalles:

- Que el estudio representa una investigación.
- El propósito del estudio.
- Los tratamientos del estudio y la probabilidad de asignación aleatoria para cada tratamiento.
- Los procedimientos a seguir en el estudio, incluyendo todos los procedimientos invasivos.

- Las responsabilidades del participante.
- Los riesgos o inconvenientes razonablemente previsibles para el participante.
- Que la participación del participante en el estudio es voluntaria y que el participante puede negarse a participar o retirarse del estudio en cualquier momento.
- Que los monitores, auditores, comité ético y las autoridades competentes tendrán acceso directo a la historia clínica original del participante para la verificación de los procedimientos o datos del estudio, sin violar la confidencialidad del participante, dentro de lo permitido por la normativa pertinente y que, al firmar el consentimiento informado, el participante o su representante legal están autorizando el acceso a estos datos
- Que los registros que identifican al participante serán confidenciales y, según lo permitido por las leyes o regulaciones pertinentes, no estarán a disposición pública, Si se publican los resultados del estudio, la identidad del participante será confidencial.
- Que se informará al participante en todo momento si se dispone de nueva información que pueda modificar su decisión de continuar en el estudio.
- Las circunstancias o razones previsibles bajo las cuales puede finalizar la participación del participante en el estudio.
- La duración esperada de la participación del participante en el estudio.

El investigador presentará un modelo de consentimiento informado apropiadamente realizado, por escrito, en cumplimiento con la BPC según las directrices de la ICH y los requisitos legales locales al CEI para su revisión y aprobación antes de iniciar el estudio (ANEXO 8). Antes de introducir participante en el estudio, se revisará una copia de consentimiento informado aprobado por el CEI con el posible participante, y se firmará y fechará. El investigador proporcionará una copia del formulario de consentimiento informado firmado de cada participante y conservará una copia en el archivo del estudio del sujeto.

9.3. Confidencialidad y acceso a los datos

Toda la información recogida será tratada de manera estrictamente confidencial, de acuerdo a la normativa vigente (Reglamento (UE) 2016/679 del Parlamento Europeo y del Consejo, de 27 de abril de 2016 (GDPR), Ley Orgánica 3/2018, de 5 de diciembre, de Protección de Datos Personales y garantía de los derechos digitales Ley 41/2002 de Autonomía del Paciente, Ley 14/1986 General de Sanidad y Ley 14/2007 de Investigación Biomédica).

La confidencialidad de los datos personales de los sujetos se mantendrá, aunque sujeta a la necesidad, por parte del monitor, de verificar los datos originales frente a la historia clínica del sujeto. En el cuaderno de recogida de datos electrónico y en toda la correspondencia del estudio figurará tan solo el código del paciente, que consistirá en un número indicativo del centro seguido de un número de dos dígitos que se asignará por orden de inclusión. La correspondencia entre la identidad del paciente y este código será conservada en un documento aparte y custodiada por el equipo investigador. Toda la información revelada por el promotor al investigador será tratada de manera estrictamente confidencial. El investigador solo hará uso de esta información para el estudio que se describe en este protocolo. Se compromete, además, a no revelar dicha información a terceros, salvo a otros colegas o empleados que participen en la ejecución del estudio y que se hallen asimismo vinculados por las obligaciones de confidencialidad.

10. CONSIDERACIONES PRÁCTICAS

10.1. Responsabilidad de todos los participantes en el ensayo

- Investigador: Los investigadores se atendrán a las normas de Buena Práctica Clínica y conocerán y seguirán los procedimientos del protocolo. Toda la información recogida durante la realización del ensayo se anotará directamente en el cuaderno de recogida de datos. Cuando se haga una corrección se deberá anotar la fecha y las iniciales de la persona que la realiza.

- Personal Auxiliar: El personal auxiliar seguirá las instrucciones dadas por el investigador en cuanto a las extracciones de muestras de sangre, su manejo y demás exploraciones complementarias.
- Promotor: Será responsable de velar por el cumplimiento de las normas legales pertinentes y de suministrar la medicación en estudio.

10.2. Desviaciones del protocolo

El equipo investigador no deberá realizar ninguna desviación ni modificación del protocolo sin el permiso del promotor, y la revisión previa y dictamen favorable por escrito a la modificación del comité ético, salvo cuando sea necesario reducir un riesgo inminente par laos participantes o cuando la modificación implique solamente aspectos logísticos o administrativos.

En ese caso, el investigador, o una persona designada por él, deberá documentar y explicar cualquier desviación del protocolo aprobado, y tan pronto como sea posible se deberá presentar al promotor para su conformidad.

10.3. Enmiendas al protocolo

No se registran enmiendas en el protocolo.

10.4. Aceptación del investigador

El compromiso de investigador está incluido en la documentación presentada al CEI.

10.5. Compromiso de publicación de resultados

Queda reflejado en el modelo de contrato firmado entre Nektium Pharma S.L. y Kinetic Performance S.L.

11. REFERENCIAS

Las referencias específicas a los cuestionarios, variables y procedimientos utilizados en este estudio se recogerán en el informe final.

12. ANEXOS

ANEXO 1. INFORMACIÓN PRODUCTO.

ANEXO 2. ESCALA DE ANSIEDAD DE HAMILTON (HAM-A).

ANEXO 3. TEST PROFILE OF MOOD STATES (POMS).

ANEXO 4. ÍNDICE DE CALIDAD DE SUEÑO DE PITTSBURGH (PSQI).

ANEXO 5. CUESTIONARIO DE EVALUACIÓN DEL SUEÑO DE LEEDS (LSEQ).

ANEXO 6. DIARIO DEL PACIENTE.

ANEXO 8. APROBACIÓN DEL COMITÉ ÉTICO DE INVESTIGACIÓN.

ANEXO 8. CONSENTIMIENTO INFORMADO.

COD: AME_HCT_2023

ANEXO: *Aframomun melegueta*

INVESTIGADORES RESPONSABLE EN NEKTIUM:
Dr. Laura López Ríos

EXTRACTO DE AFRAMOMUM MELEGUETA

- Extracto hidroalcohólico al 70% de las semillas
- Estandarizado a gingerosidos totales.
- Declaración de alérgenos (Directiva 2007/68/CE): A pesar de que no se ha descrito presencia de alérgenos conocidos en la literatura, no se descartan posibles reacciones alérgicas en personas sensibles a la capsaicina o gingeroles.
- Este producto no procede de organismos modificados genéticamente (no-GMO).
- Es apto para el consumo humano.

AFRAMOMUM MELEGUETA

El *Aframomum melegueta* (AM) es una especie herbácea de la familia de los Zingiberaceas, conocido comúnmente como “granos del paraíso” o “pimienta melegueta”. Se trata de una planta herbácea rizomatosa aromática y perenne cuya semilla se usa normalmente como condimento o especia culinaria.

USO TRADICIONAL DE LA SEMILLA

Al margen de su uso culinario tradicional (como especia o añadida en bebidas), los granos del paraíso también han sido usados en el Este de África para tratar resfriados y dolores de garganta o entrar en calor en días fríos (masticados) Las semillas masticadas o en decocciones también se han utilizado para tratar la disentería, dolor abdominal, estreñimiento, reumatismo, inflamación, mordedura de serpiente e hipertensión. Mezcladas con sal y colocadas en el

interior de la boca se han empleado para tratar alteraciones del sueño. En hombres se han usado en combinación con otras plantas para tratar la infertilidad. En animales, las semillas impregnadas en alcohol se administran a perros de caza como excitante (Trinidad) (1)(2)(3).

COMPOSICIÓN NUTRICIONAL

La vitamina en mayor concentración es la vitamina C (12.3 mg/100g) pero también tiene tiamina (Vit B1), Riboflavina y ácido nicotínico. El mineral presente en mayor concentración es el hierro (1.8 mg/100g) y además contiene magnesio, calcio, fósforo, sodio, zinc, cobre y manganeso. Contiene piperina, un alcaloide que se encuentra principalmente en la pimienta negra y que le confiere su sabor amargo (4).

El contenido total de polifenoles varía de 2-2.8%, principalmente taninos y flavoniodes (como el quercetin, el kaemferol y el ácido hidroxibenzoico). Los compuestos activos de mayor relevancia en el *Aframomum melegueta* se encuadran un grupo conocido como gingeroles, que incluye el 6-gingerol (1-1.6%) seguido del 6-paradol, 7-paradol y 6-shogaol (4).

PROPIEDADES Y ESTUDIOS CIENTÍFICOS

Se han realizado estudios tanto in vitro como in vivo sobre sus propiedades y se ha visto que es un buen *antioxidantes*, 400 gr de semilla tienen un efecto similar la vitamina E (5), su efecto puede ser dosis dependiente (6) y que podría modificar la respuesta de las células blancas frente a daño tisular (7). Tiene capacidad *antiinflamatoria* ya que inhibe la producción de prostaglandinas y leucotrienos así como de la óxido nítrico sintasa (NOS) y la COX2 (Ciclooxigenasa 2) (in vitro) (6) así como la expresión de genes pro-inflamatorios (1). También se ha observado un efecto *anti-estresante* al inhibir el receptores beta-adrenergicos (8), (9), anti-estrogénico (10) y *neuroprotector* (en modelos animales de daño cerebral por traumatismo (TBI) un extracto de aframomum redujo la neurodegeneración en los supervivientes de TBI y, en algunos casos restauraba la expresión de genes (11). Un estudio reciente desarrollado en ratas evaluó el efecto beneficioso de un extracto alcohólico de AM en daño cognitivo inducido por scopolamina (12)

A nivel de homeostasis metabólica, el extracto de semillas y el 6-paradol (presente en el extracto alcohólico de las semillas), puede incrementar la temperatura de la grasas parda y reduce la grasa visceral, por lo que se considera un *termogénico* (13). En modelos de diabetes tipo 2 de ratones

(tratados con alloxan) los niveles de glucemia tuvieron una reducción mayor en animales tratados con un extracto acuoso de las semillas que los tratados con metformina (14). Además reduce los niveles de colesterol, la actividad de la ACE y las enzimas pancreáticas (*hepatoprotector*) sobre ratas hipercolesterolémicas y que dichos efectos podrían estar modulados por su contenido en aminoácidos (tales como GABA, tirosina, fenilalanina y triptófano combinados con los fitoquímicos antioxidantes que presenta (15).

Ejerce un efecto *anti-ulceroso* e incrementa la resistencia de la mucosa gástrica (16). Tanto los extractos alcohólico como acuoso puede actuar como anti-fúngico o anti-bacteriano frente a bacterias gram positivas y gram negativas (como por ejemplo *E. coli, Pseudomonas, Shigella spp*. o *Klebsiella)* (17)(16). Y se ha observado una mejora hepática en ratas con daño hepático inducido tras la administración de extractos alcohólicos o de polvo de semillas disuelto en agua (18).

En modelos de animales machos el extracto de AM mejora la erección e incrementa tanto el volumen como la frecuencia de eyaculación (1),(19), reduce los niveles de PSA (Prostate Specific Antigen) e incrementaba los niveles de testosterona (20).

ENSAYOS CLÍNICOS EN HUMANO

Potenciador de fármacos: Estudios in vitro han puesto de manifiesto la capacidad del extracto acuoso y del extracto alcohólico de *A. melegueta* para inhibir CYP3A4, CYP3A5 y CYP3A7, interfiriendo así con el metabolismo de algunos fármacos (*in vitro*) (21).

Hipertensión arterial: Estudio en sujetos normotensos e hipertensos (n=18) la ingesta de 10-20 semillas (1.2mg/grano) de granos del paraíso pareció ejercer un efecto positivo sobre los niveles de tensión arterial tanto en sujetos normotensos como hipertensos (22).

Gasto de energía total (EE): Estudio de imagen de la grasa parda (PET) con 19 participantes jóvenes (hombres) y sanos a los que se les administró 40 mg de extracto alcohólico de AM. Del estudio se concluyó que el extracto de AM incrementó la actividad del tejido adiposo pardo lo que llevó a un incremento del gasto energético total. En otro estudio la administración de 30 mg del extracto de AM a 19 jóvenes (mujeres) durante 4 semanas dio como resultado una reducción del perímetro de cintura a nivel abdominal. En ambos estudios no refieren haber observado no efectos secundarios ni molestias derivadas de la ingesta del extracto de AM (23)(24).

Afección ocular: Tras la administración de una única dosis de 350 mg de semillas de AM a 10 hombres de 30-35 años, sanos y normopesos se observó un incremente del punto de convergencia (17.2%) y una reducción en la amplitud de acomodación acular (9.2%) que se traducen en visión doble y borrosa de manera transitoria (25).

Calidad de vida de mujeres pre-menopaúsicas: Tras la administración de una dosis de 50mg/día, incluida en una formulación combinada con otros dos ingredientes, durante 8 semanas, en un total de 57 mujeres premenopáusicas no se manifestaron efectos secundarios y sí una mejora en la puntuación de la escala de calidad de vida específico para este colectivo.

SEGURIDAD Y TOXICIDAD

Estudios de fase aguda: La administración en ratas de un extracto alcohólico de semillas de AM en un rango de 0.25-4g/kg de peso no se asoció con síntomas tóxicos no con mortalidad (periodo de observación de 14 días) (16).

Estudios en fase crónica: Tras la administración de un extracto acuoso de AM no se observó cambios histológicos en la glándula adrenal aunque sí ganancia de peso. Por su parte, la administración de un extracto alcohólico de AM (26).

Hepatotoxicidad: Estudio desarrollado en ratas a las que se le administraron un extracto metanólico de 300 mg/kg de peso y se evaluó la la exposición sub-crónica a 7, 14 y 21 días. La toxicidad hepática se evaluó en relación a niveles alterados de enzimas hepáticas circulantes en suero. Los niveles de AST (aspartato aminotransaminasa) se incrementa progresivamente. Los niveles de ALT (fosfatasa alcalina), de ALP (alanina aminotransferasa) y los niveles de bilirrubina totales se elevaron ligera y significativamente a los 14 y 21 días. Los niveles de albúmina en suero no sufrieron cambios en ese periodo. La histopatología de hígado reveló una necrosis media de manera local a los 7 días, multifocal moderada a los 14 días y severa a los 21. Por lo que a dosis de 300 mg/kg de animal, el extracto metanólico de AM induce toxicidad hepática (27). Por el contrario, el extracto acuoso o el extracto con un 45% de alcohol parecemn ejercer un efecto beneficioso a nivel hepático (hepatoprotector) gracias a su capacidad antioxidante a nivel hepático (18)

EMBARAZO Y CONCEPCIÓN

Estudios en ratas han demostrado que la administración intraperitoneal de un extracto acuoso de las semillas reducía la ganancia de peso durante la gestación. También se le atribuyen efecto abortivo a dosis de elevadas (4mg/kg de peso corporal) (28).

Por ello no es aconsejable su administración a dosis elevadas a poblaciones femeninas fértiles y en disposición de tener descendencia.

ESTADO REGULATORIO

La semilla del *Aframomum melegueta* está incluida como "alimento" en la lista BelFrIt (Lista harmonizada de suplementos alimenticios de origen botánicos acordada por Bélgica, Francia e Italia).

La Comisión Europea, en el catálogo de "Novel Food" considera las semillas aptas para el consumo ya que se encontraban en el mercado como alimento o ingrediente alimentario y se consumían en gran medida antes del 15 de mayo de 1997. Por lo tanto, su acceso al mercado no está sujeto al Reglamento (CE) Nº 258/97 sobre nuevos alimentos

Aframomum melegueta, también conocido como granos del paraíso, se menciona en el Code of Federal Regulations (reglamento de la FDA, USA) Title 21 "21 CFR – PART 182 – SUBSTANCES GENERALLY RECOGNIZED AS SAFE – Sec. 182.10, sección que identifica para el consumo humano a especias y otros aromatizantes. Además, por estar listado en el United Natural Products Alliance, el "the Herbs of Commerce, first edition (1992) y el "The american Herbal Products Association´s Botanical Safety Handbook (1997) es considerado como un "Old Dietary Ingrediente" y apto para el consumo humano

La Farmacopea africana (African Herbal Pharmacopoeia) recoge la información que describe a la planta, parte de uso, usos ethofarmacológicos y su composición.

PRESENCIA EN EL MERCADO

PATENTE

La Oficina Europea de Patentes recoge al menos tres patentes que incluyen al *Aframomum melegueta* para distintas aplicaciones

PRODUCTO FINAL

El **Parodoxine™** es un producto a base de extracto de granos del paraíso estandarizado a un 12-15% de gingeroles. Ha sido desarrollado por GENABOLIX, orientado a la quema de grasa y pérdida de pesos. Se recomienda una dosis de 40mg/servicio.

También podemos encontrar extracto de AM como parte de la composición de productos destinados a deporte para potenciar la quema de grasas y la termogénesis (Ejemplos: After burm, de la casa “Magnum Nutraceutical”, Diablo™ de la casa “ANSperformance”, etc) a dosis de 40mg por cápsula y en los que se recomiendan tomar tres cápsulas por día.

También se puede encontrar en el mercado como genérico sin marca registrada estandarizado a distintas concentraciones de 6-paradol (12-55%), tanto en polvo como en extracto líquido.

REFERENCIAS

1. Umukoro S, Ashorobi RB. Further studies on the antinociceptive action of aqueous seed extract of Aframomum melegueta. J Ethnopharmacol. 2007 Feb 12;109(3):501–4.
2. Iwu MM. Handbook of African Medicinal Plants. 1º ed. London: CRC Press; 1993. 506 p.
3. Lans C, Harper T, Georges K, Bridgewater E. Medicinal and ethnoveterinary remedies of hunters in Trinidad. BMC Complement Altern Med. 2001;1:10.
4. D.O. A, K.T. O, T.P. O, O.J. O, I.A A. Nutritional, Minerals and Phytochemicals composition of Garcinia cola [Bitter cola] and Aframomum melegueta [Alligator pepper]. J Environ Sci Toxicol Food Technol. 2014;8(1):8 6–91.
5. Adegoke GO, Makinde O, Falade KO, Uzo-Peters PI. Extraction and characterization of antioxidants from Aframomum melegueta and Xylopia aethiopica. Eur Food Res Technol. 2003 Jun 22;216(6):526–8.
6. Dugasani S, Pichika MR, Nadarajah VD, Balijepalli MK, Tandra S, Korlakunta JN. Comparative antioxidant and anti-inflammatory effects of [6]-gingerol, [8]-gingerol, [10]-gingerol and [6]-shogaol. J Ethnopharmacol. 2010 Feb 3;127(2):515–20.
7. Umukoro S, Ashorobi BR. Further pharmacological studies on aqueous seed extract of Aframomum melegueta in rats. J Ethnopharmacol. 2008 Feb 12;115(3):489–93.
8. Ahounou JF, Ouedraogo GG, Gbenou JD, Ouedraogo S, Agbodjogbe WK, Dansou PH, et al. Spasmolytic effects of aqueous extract of mixture from Aframomumum melegueta (K Schum) - Citrus aurantifolia (Christm and Panzer) on isolated trachea from rat. African J Tradit Complement Altern Med AJTCAM. 2012;9(2):228–33.
9. Umukoro S, and Ashorobi R. Anti - Stress Po tential of Aqueous Seed Extract of Aframomum Melegueta. African J Biomed Res. 2005;8:11 9–1 21.
10. Ali M. El-Halawanya MH. Anti-oestrogenic diarylheptanoids from Aframomum melegueta with in silico oestrogen receptor alpha binding conformation similar to enterodiol and enterolactone. Food Chem. 2012;134(1):219–26.
11. Kumar A. Neuroprotective Effects of Aframomum melegueta Extract after Experimental Traumatic Brain Injury. Nat Prod Chem Res. 2015;3(1).
12. Ishola IO, Awoyemi AA, Afolayan GO. Involvement of Antioxidant System in the

Amelioration of Scopolamine-Induced Memory Impairment by Grains of Paradise (Aframomum melegueta K. Schum.) Extract. Drug Res (Stuttg). 2016 Sep;66(9):455–63.
13. Iwami M, Mahmoud FA, Shiina T, Hirayama H, Shima T, Sugita J, et al. Extract of grains of paradise and its active principle 6-paradol trigger thermogenesis of brown adipose tissue in rats. Auton Neurosci. 2011 Apr 26;161(1-2):63–7.
14. Adesokan Ayoade A* AMA and AGS. Evaluation of Hypoglycaemic Efficacy of Aqueous Seed Extract of Aframomum melegueta in Alloxan-induced Diabetic Rats. Sierra Leone J Biomed Res. 2010;2(2):91–4.
15. Adefegha SA, Oboh G, Adefegha OM, Henle T. Alligator pepper/Grain of Paradise (Aframomum melegueta) modulates Angiotensin-I converting enzyme activity, lipid profile and oxidative imbalances in a rat model of hypercholesterolemia. Pathophysiol Off J Int Soc Pathophysiol. 2016 Sep;23(3):191–202.
16. S. Rafatullah, A. M. Galal MAA-Y& MSA-S. Gastric and duodenal ulcer and cytoprotective effects of Aframomum melegueta in rats. Int J Pharmacogn. 1995;33(4):311–6.
17. Konning GH, Agyare C, Ennison B. Antimicrobial activity of some medicinal plants from Ghana. Fitoterapia. 2004 Jan;75(1):65–7.
18. Nwozo SO, Oyinloye BE. Hepatoprotective effect of aqueous extract of Aframomum melegueta on ethanol-induced toxicity in rats. Acta Biochim Pol. 2011;58(3):355–8.
19. Mbongue GYF, Kamtchouing P, Dimo T. Effects of the aqueous extract of dry seeds of Aframomum melegueta on some parameters of the reproductive function of mature male rats. Andrologia. 2012 Feb;44(1):53–8.
20. Akpanabiatu MI, Ekpo ND, Ufot UF, Udoh NM, Akpan EJ, Etuk EU. Acute toxicity, biochemical and haematological study of Aframomum melegueta seed oil in male Wistar albino rats. J Ethnopharmacol. 2013 Nov 25;150(2):590–4.
21. Agbonon A, Eklu-Gadegbeku K, Aklikokou K, Gbeassor M, Akpagana K, Tam TW, et al. In vitro inhibitory effect of West African medicinal and food plants on human cytochrome P450 3A subfamily. J Ethnopharmacol. 2010 Mar 24;128(2):390–4.
22. . BASL, . AOA, . GAE, . ADE. Hypotensive and Antihypertensive Effects of Aframomum melegueta Seeds in Humans. Int J Pharmacol. 2007 Apr 1;3(4):311–8.
23. Sugita J, Yoneshiro T, Hatano T, Aita S, Ikemoto T, Uchiwa H, et al. Grains of paradise (Aframomum melegueta) extract activates brown adipose tissue and increases whole-body energy expenditure in men. Br J Nutr. 2013 Aug;110(4):733–8.
24. Sugita J, Yoneshiro T, Sugishima Y, Ikemoto T, Uchiwa H, Suzuki I, et al. Daily ingestion of grains of paradise (Aframomum melegueta) extract increases whole-body energy expenditure and decreases visceral fat in humans. J Nutr Sci Vitaminol (Tokyo). 2014;60(1):22–7.
25. Igwe SA, Emeruwa IC, Modie JA. Ocular toxicity of Afromomum melegueta (alligator pepper) on healthy Igbos of Nigeria. J Ethnopharmacol. 1999 Jun;65(3):203–6.
26. Obike H I, Ezejindu D N AA. The effects of Aframomum melegueta aqueous extract on the adrenal gland of adult wistar rats. Int J Res Med Heal Sci. 2014;3(6):1–6.
27. Ilic N, Schmidt BM, Poulev A, Raskin I. Toxicological evaluation of grains of paradise (Aframomum melegueta) [Roscoe] K. Schum. J Ethnopharmacol. 2010 Feb 3;127(2):352–6.
28. Inegbenebor U, Ebomoyi MI, Onyia KA, Amadi K, Aigbiremolen AE. Effect of aqueous extract of alligator pepper (Zingiberaceae aframomum melegueta) on gestational weight gain. Niger J Physiol Sci. 2009 Dec;24(2):165–9.

SOPORTE REGULATORIO

Aframomum melegueta is listed in (see below):

- The Old Dietary Ingredient list, 2011, UNPA
- The Herbs of Commerce, First edition, 1992, AHPA
- The American Herbal Products Association's Botanical Safety Handbook 1997

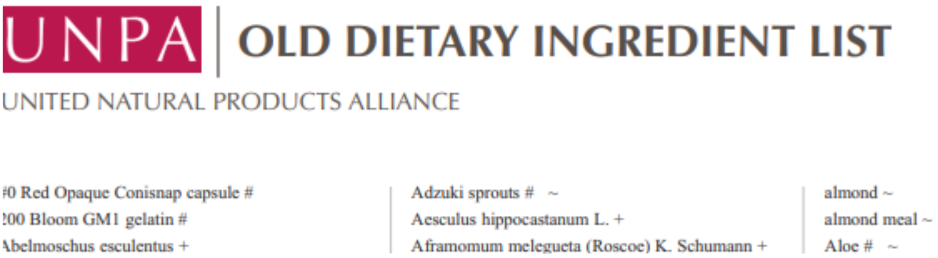

UNPA | OLD DIETARY INGREDIENT LIST

UNITED NATURAL PRODUCTS ALLIANCE

#0 Red Opaque Conisnap capsule #
200 Bloom GM1 gelatin #
Abelmoschus esculentus +
Adzuki sprouts # ~
Aesculus hippocastanum L. +
Aframomum melegueta (Roscoe) K. Schumann +
almond ~
almond meal ~
Aloe # ~

Figura 1: Image from "Old Dietary Ingredient List (UNPA)".

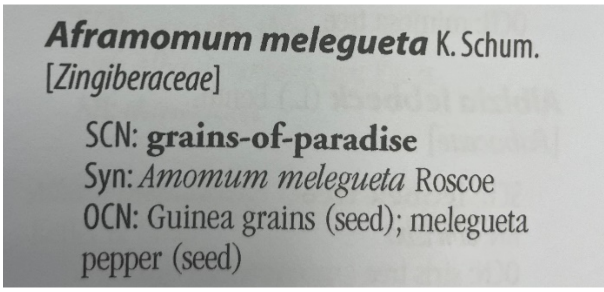

Aframomum melegueta K. Schum.
[*Zingiberaceae*]
SCN: **grains-of-paradise**
Syn: *Amomum melegueta* Roscoe
OCN: Guinea grains (seed); melegueta pepper (seed)

Figura 2: Image from "Herbs of commerce".

Moreover, Aframomum melegueta Rosc. ("Grains of paradise") synonym of Aframomum melegueta K. schum, is mentioned in the Code of Federal Regulations Title 21 "21 CFR – PART 182 – SUBSTANCES GENERALLY RECOGNIZED AS SAFE – Sec. 182.10 Spices and other natural seasonings and flavorings". Aframomum melegueta is used as spice and is generally recognized as safe for its intended use.

21 CFR Part 182 (up to date as of 10/06/2022)
Substances Generally Recognized as Safe

Common name	Botanical name of plant source
Camomile (chamomile), German or Hungarian	Matricaria chamomilla L.
Capers	Capparis spinosa L.
Capsicum	Capsicum frutescens L. or Capsicum annuum L.
Caraway	Carum carvi L.
Caraway, black (black cumin)	Nigella sativa L.
Cardamom (cardamon)	Elettaria cardamomum Maton.
Cassia, Chinese	Cinnamomum cassia Blume.
Cassia, Padang or Batavia	Cinnamomum burmanni Blume.
Cassia, Saigon	Cinnamomum loureirii Nees.
Cayenne pepper	Capsicum frutescens L. or Capsicum annuum L.
Celery seed	Apium graveolens L.
Chervil	Anthriscus cerefolium (L.) Hoffm.
Chives	Allium schoenoprasum L.
Cinnamon, Ceylon	Cinnamomum zeylanicum Nees.
Cinnamon, Chinese	Cinnamomum cassia Blume.
Cinnamon, Saigon	Cinnamomum loureirii Nees.
Clary (clary sage)	Salvia sclarea L.
Clover	Trifolium spp.
Coriander	Coriandrum sativum L.
Cumin (cummin)	Cuminum cyminum L.
Cumin, black (black caraway)	Nigella sativa L.
Elder flowers	Sambucus canadensis L.
Fennel, common	Foeniculum vulgare Mill.
Fennel, sweet (finocchio, Florence fennel)	Foeniculum vulgare Mill. var. duice (DC.) Alex.
Fenugreek	Trigonella foenum-graecum L.
Galanga (galangal)	Alpinia officinarum Hance.
Geranium	Pelargonium spp.
Ginger	Zingiber officinale Rosc.
Grains of paradise	Amomum melegueta Rosc.

Image 3: Image from “Code of Feneral Regulations”.

nektium

SPECIFICATION SHEET

Product name: *Aframomum melegueta* extract 7-10% total gingerols
Common name: **Alligator pepper**
Code: AME07S

01 Product description	Specification	Test method
Plant part used	Seeds	Visual
Botanical name	*Aframomum melegueta*	Macroscopic
Carrier(s) used	Arabic gum: citrus pectin: pea protein (2:1:1; w:w:w)	≥ 67%
Plant: Extract ratio	5-8:1	By weight

02 Physical data	Specification	Test method
Appearance	Free flowing powder	Visual
Color	Light brown	Visual
Aroma	Characteristic	Organoleptic
Flavor	Spicy, characteristic	Organoleptic
Particle Size	100 % Through 80 Mesh	US standard sieve
Solubility in Water	Practically insoluble	EU Pharm. 5.11.
Bulk Density	0.3-0.5 (g/cm^3)	USP 616 / EU Pharm. 2.9.34.

03 Chemical data	Specification	Test method
Total gingerols (capsaicin eq.)	7.0% - 10.0 %	UPLC (MUPLCXX)
Moisture	≤ 7.0 %	USP 921
Total heavy metal	TBD	EU Pharmacopeia 2.4.8.
Total arsenic	TBD	ICP-MS
Lead	TBD	ICP-MS
Cadmium	TBD	ICP-MS
Mercury	TBD	ICP-MS
Residual solvents	TBD	GC-MS (USP 467 / Directive (EU)2009/32 and 2016/1855)
Pesticides	Complies with USP 565 and Regulation 396/2005/CE and [illegible]	
Contaminants	Complies with Regulation EC 1881/2006 and amendments	

04 Microbiological data	Specification	Test method
Total plate count	TBD	ISO 4833

Nektium Pharma S.L.
C/ Las Mimosas 8
35118 Agüimes
Las Palmas, España

nektium.com
info@nektium.com
+34 928 734 132

Yeast & mold	TBD	ISO 7954
Salmonella	TBD	ISO 6579
E. coli	TBD	EU Pharm. 2.6.13.
Total Coliforms	TBD	M0042 - Part V. VRB Plate Count
Staphylococcus aureus	TBD	EU Pharm. 2.6.13.

05 Additional information

Extraction method	Water: ethanol (30:70) extraction and spray dried
Packing	25 kg HDPE drums with double PE bags
Storage	Original container in a cool, dry place
Shelf life	TBD years (currently under evaluation)
Country of origin / manufactured	Spain
Intended use	Nutraceutical
Non-Allergens/Gluten	This product does not contain any of the food allergens cited in the Regulation (EU) No 1169/2011
Non-BSE/TSE	All ingredients in the product are of vegetable origin
Non-Irradiation	This material has not been subjected to irradiation
Nanomaterial	This material is not produced using Nanotechnology and is free from Nanoparticles
Non-GMO status	This product is neither considered genetically modified nor derived from any genetically modified organisms, as defined by the EC regulations 1830/2003/EC, 1829/2003/EC and any amending legislation

Ver. draft
31/01/2022

DRAFT

nektium

/// Specification Sheet

DRAFT VERSION

/ C/ Las Mimosas 8, 35118 Agüimes. Las Palmas. Spain.

Nektium Pharma S.L.
C/ Las Mimosas 8, Pol. Ind. Arinaga
35118 · Agüimes, Las Palmas. Spain

nektium.com
quality@nektium.com
+34 928 734 132

HAMILTON ANXIETY RATING SCALE (HAM-A)

Reference: Hamilton M. (1959). The assessment of anxiety states by rating. Br J Med Pyschol, 32, 50-55.

A continuación se presentan una series de frases que se refieren a las reacciones que la gente comúnmente manifiesta cuando se enfrenta a situaciones de la vida que se toman conflictivas. Lea cuidadosamente le cuestionario y procure identificar con una X las reacciones que han aparecido en usted:

0) Nunca

1) Algunas veces

2) Varias veces

3) Casi siempre

4) Siempre

CUESTIONARIO		RESPUESTAS				
1	Presiento que algo malo puede pasarme, que me puede suceder lo pero. Me siento preocupado, irritable.	0	1	2	3	4
2	Tengo la sensación de inquietud y no puedo relajarme. Me canso fácilmente. Me sobresalto. Tiemblo. Lloro con facilidad.					
3	Tengo miedo a la oscuridad, de quedarme solo, de la gente desconocida, de los animales, del tráfico, de la multitud.					
4	Tengo dificultad para conciliar el sueño. Me despertó varias veces. Me levanto cansado. No hay sueño reparador.					
5	Tengo mala memoria, especialmente de los eventos recientes. Me cuesta concentrarme.					
6	No tengo interés por lo que me rodea. Me siento triste. Me adormezco por el día y no puedo dormirme por la noche.					
7	Siento dolores y molestias musculares. Me rechinan los dientes. Tengo la voz poco firme e insegura.					
8	Tengo zumbidos de odio. Vista borrosa. Siento oleadas de calor o frío. Tengo picores y sensación de debilidad.					
9	Siento que mi corazón late más deprisa que de costumbre. Siento palpitaciones, dolores en el pecho, agitación.					
10	Tengo sensación de ahogo y falta de aire. Necesito respirar. Siento en el pecho una opresión o pena.					
11	Tengo dificultad al tragar. Siento ardores, pesadez o sensación de plenitud gástrica. Siento náuseas y vómitos. Estreñimiento.					
12	Siento necesidad de orinar frecuentemente, aunque sea poco. Tengo alteraciones hormonales. Mi vida sexual está deteriorada.					
13	Siento la boca seca. Me ruborizo y padezco fácilmente. Me mareo y las cosas me dan vueltas. Se me ponen los pelos de punta.					
14	Me he sentido incómodo, inquieto, tenso, impaciente, me sudan las manos y el pulso se me acelera al contestar estas preguntas.					

PROTOCO PARA LA APLICACIÓN DEL TEST POMS.

NOMBRE: ___________________________ FECHA: _____________

FRENTE A CADA ASPECTO INDIQUE SEGÚN LA ESCALA LA RESPUESTA QUE USTED CONSIDERE. RECUERDE VERIFICAR QUE CADA ASPECTO ESTE RESPONDIDO ASEGURESE DE SOLO RESPONDER CON UN SOLO INDICADOR.

Lea atentamente la lista de palabras que se describen en el siguiente cuestionario, estas palabras describen sentimientos que tienen las personas. Después de leer cada palabra fíjese en las cinco opciones que aparecen arriba y elija entre ellas la que mejor describa cómo se ha sentido usted durante las últimas 24 horas. Seleccione un valor en la casilla situada a la derecha de cada palabra. No deje ninguna casilla en blanco.

- PREGUNTAS

1. Tenso	20.Fatigado
2. Inestable	21.Exhausto
3. Con los nervios de punta	22.Perezoso
4. Asustado	23.Abatido
5. Relajado	24.Tirado
6. Intranquilo	25.Infeliz
7. Inquieto	26.Arrepentido por cosas hechas
8. Nervioso	27.Triste
9. Ansioso	28.Melancólico
10.Animado	29.Falto de esperanza
11.Activo	30.Indigno
12.Energético	31.Desanimado
13.De buen humor	32.Solo
14.Alerta	33.Miserable
15.Lleno de dinamismo	34.Pesimista
16.Despreocupado	35.Desesperado
17.Vigoroso	36.Inútil
18.Rendido	37.Aterrorizado
19.Apático	38.Culpable

NADA				POCO			MODERADO				BASTANTE			MUCHÍSIMO			
0				1			2				3			4			
	0	1	2	3	4		0	1	2	3	4		0	1	2	3	4
1. Tenso						17. Vigoroso						33. Miserable					
2. Inestable						18. Rendido						34. Pesimista					
3. Con los pelos de punta						19. Apático						35. Desesperado					
4. Asustado						20. Fatigado						36. Inútil					
5. Relajado						21. Exhausto						37. Aterroizado					
6. Intranquilo						22. Perezoso						38. Culpable					
7. Inquieto						23. Abatido											
8. Nervioso						24. Tirado (muy cansado)											
9. Ansioso						25. Infeliz											
10. Animado						26. Arrepentido por cosas hechas											
11. Activo						27. Triste											
12. Energético						28. Melancólico											
13. De buen humor						29. Falto de esperanza											
14. Alerta						30. Indigno											
15. Lleno de dinamismo						31. Desanimado											
16. Despreocupado						32. Solo											

Cuestionario de Pittsburg de Calidad de sueño.

Nombre: Fecha:

1. Durante el último mes, ¿cuál ha sido, normalmente, su hora de acostarse?

2. ¿Cuánto tiempo habrá tardado en dormirse, normalmente, las noches del último mes?
   1. Menos de 15 min.
   2. Entre 16-30 min.
   3. Entre 31-60 min.
   4. Más de 60 min.

3. Durante el último mes, ¿a qué hora se ha levantado habitualmente por la mañana?

4. ¿Cuántas horas calcula que habrá dormido verdaderamente cada noche durante el último mes?
5.

5. Durante el último mes, cuántas veces ha tenido usted problemas para dormir a causa de:<br>a) No poder conciliar el sueño en la primera media hora:	1. Ninguna vez en el último mes<br>2. Menos de una vez a la semana<br>3. Una o dos veces a la semana<br>4. Tres o más veces a la semana
b) Despertarse durante la noche o de madrugada	1. Ninguna vez en el último mes<br>2. Menos de una vez a la semana<br>3. Una o dos veces a la semana<br>4. Tres o más veces a la semana
c) Tener que levantarse para ir al servicio	1. Ninguna vez en el último mes<br>2. Menos de una vez a la semana<br>3. Una o dos veces a la semana<br>4. Tres o más veces a la semana
d) No poder respirar bien	1. Ninguna vez en el último mes<br>2. Menos de una vez a la semana<br>3. Una o dos veces a la semana<br>4. Tres o más veces a la semana
e) Toser o roncar ruidosamente	1. Ninguna vez en el último mes<br>2. Menos de una vez a la semana<br>3. Una o dos veces a la semana<br>4. Tres o más veces a la semana

f) Sentir frío	1. Ninguna vez en el último mes<br>2. Menos de una vez a la semana<br>3. Una o dos veces a la semana<br>4. Tres o más veces a la semana
g) Sentir demasiado calor	1. Ninguna vez en el último mes<br>2. Menos de una vez a la semana<br>3. Una o dos veces a la semana<br>4. Tres o más veces a la semana
h) Tener pesadillas o malos sueños	1. Ninguna vez en el último mes<br>2. Menos de una vez a la semana<br>3. Una o dos veces a la semana<br>4. Tres o más veces a la semana
h) Sufrir dolores	1. Ninguna vez en el último mes<br>2. Menos de una vez a la semana<br>3. Una o dos veces a la semana<br>4. Tres o más veces a la semana
i) Otras razones.	1. Ninguna vez en el último mes<br>2. Menos de una vez a la semana<br>3. Una o dos veces a la semana<br>4. Tres o más veces a la semana
6. Durante el último mes, ¿cómo valoraría en conjunto, la calidad de su sueño?	1. Muy buena<br>2. Bastante buena<br>3. Bastante mala<br>4. Muy mala
7. Durante el último mes, cuántas veces habrás tomado medicinas (por su cuenta o recetadas por el médico) para dormir?	1. Ninguna vez en el último mes<br>2. Menos de una vez a la semana<br>3. Una o dos veces a la semana<br>4. Tres o más veces a la semana
8. Durante el último mes, ¿cuántas veces ha sentido somnolencia mientras conducía, comía o desarrollaba alguna otra actividad?	1. Ninguna vez en el último mes<br>2. Menos de una vez a la semana<br>3. Una o dos veces a la semana<br>4. Tres o más veces a la semana
9. Durante el último mes, ¿ha representado para usted mucho problema el tener ánimos para realizar alguna de las actividades detalladas en la pregunta anterior?	1. Ningún problema<br>2. Sólo un leve problema<br>3. Un problema<br>4. Un grave problema
10. ¿Duerme usted solo o acompañado?	1. Solo<br>2. Con alguien en otra habitación<br>3. Misma habitación, pero otra cama<br>4. En la misma cama

LEEDS SLEEP EVALUATION QUESTIONNAIRE

Nombre:

1. MAL 2. REGULAR 3. BUENO 4. EXCELENTE

1. ¿Cómo describiría la forma en que se duerme actualmente en comparación con lo habitual?
2. ¿Cómo describiría la calidad de su sueño con comparación con el sueño normal?
3. ¿Cómo describirías tu despertar en comparación con lo habitual?
4. ¿Cómo te has sentido al despertar?
5. ¿Cómo te sientes ahora?
6. ¿Cómo describirías tu equilibrio y coordinación al levantarte?

RESPUESTAS

Pregunta		1	2	3	4	5	6
Fecha							
Fecha							
Fecha							

INFORMACIÓN DIARIA

DÍA 1

PREGUNTA	SÍ	NO	OBSERVACIONES
¿Has realizado las actividades diarias rutinarias (trabajar, comprar, etcétera) en el día de hoy?			
¿Has realizado alguna actividad diferente (excursión, cena con amigos, etcétera) en el día de hoy?			
¿Has realizado algún tipo de actividad física (gimnasio, correr, etcétera) en el día de hoy?			
¿Has realizado entre 3 y 5 comidas en el día de hoy?			
¿Has comido como mínimo 3 piezas de frutas o verduras en el día de hoy?			
¿Has tenido dolor de cabeza?			
¿Has tenido algún problema digestivo (dolor de barriga, estreñimiento, etcétera)?			
¿Te has sentido más fatigado/a o cansado/a que en otros días?			

DÍA 2

PREGUNTA	SÍ	NO	OBSERVACIONES
¿Has realizado las actividades diarias rutinarias (trabajar, comprar, etcétera) en el día de hoy?			
¿Has realizado alguna actividad diferente (excursión, cena con amigos, etcétera) en el día de hoy?			
¿Has realizado algún tipo de actividad física (gimnasio, correr, etcétera) en el día de hoy?			
¿Has realizado entre 3 y 5 comidas en el día de hoy?			
¿Has comido como mínimo 3 piezas de frutas o verduras en el día de hoy?			
¿Has tenido dolor de cabeza?			
¿Has tenido algún problema digestivo (dolor de barriga, estreñimiento, etcétera)?			
¿Te has sentido más fatigado/a o cansado/a que en otros días?			

COMITÉ DE ÉTICA DE INVESTIGACIÓN

Alicante, jueves 12 de enero de 2023

Reunidos en el Parque Científico de Alicante, el **Comité Ético de Investigación perteneciente a Kinetic Performance**, en su reunión del día 09-01-2023 (acta 02/23), evaluó el proyecto de investigación:

TÍTULO: Efectos de un extracto vegetal modulador del sistema endocanabinoide y su capacidad ansiolítica sobre personas senior en situaciones de estrés o ansiedad.

Nº de Registro: K04/023

Código del promotor: AME_HCT_2023

Investigador solicitante: Carlos Elvira Aranda

Decisión: APROBADO (09-01-23)

Este Comité Ético de Investigación, según el Artículo 5º, considera que el proyecto citado, siendo promotor Nektium, es **ético y metodológicamente aceptable. Cumpliendo así las normas internacionales BPC (CPMP/ICH/135/95)**. Así mismo, considera que los investigadores que solicitaron la evaluación de este Comité para la investigación son competentes para llevar a cabo este proyecto que está enmarcado dentro de las líneas de investigación prioritarias del Parque Científico de Alicante y Kinetic Performance.

DEPARTAMENTO DE DIDÁCTICA GENERAL
Y DIDÁCTICAS ESPECÍFICAS
UNIVERSIDAD DE ALICANTE

Fdo: Dr. José Antonio Pérez Turpin
Presidente del C.E.I.

CONSENTIMIENTO INFORMADO

D./Da____
con DNI____.

MANIFIESTO QUE:

1. He recibido de la empresa KINETIC PERFORMANCE. toda la información necesaria, de forma confidencial, clara, comprensible y satisfactoria sobre la naturaleza y propósito de los objetivos, procedimientos y temporalidad que se seguirán a lo largo del estudio científico sobre EFECTOS DE UN EXTRACTO VEGETAL SOBRE EL ESTRÉS Y ANSIEDAD gestionados por la empresa KINETIC PERFORMANCE.
2. Acepto el trato de mis datos personales, incluidos datos médicos, antropométricos y de otro tipo, para los fines del estudio tal y como se describe la Ley de protección de datos Reglamento General de Protección de Datos (Reglamento UE 2016-679 del Parlamento europeo y del Consejo, de 27 de abril de 2016) y la normativa española sobre protección de datos de carácter personal vigente
3. Así mismo, doy mi consentimiento para la divulgación de los datos relacionado con el estudio, como mi edad, sexo, o información médica personal, al promotor del estudio.
4. Acepto que se almacenen y utilicen las muestras biológicas (muestras sanguíneas) que me extraigan para los fines de este estudio.
5. Acepto que, una vez recibida toda la información necesaria, acuerdo y comprometo a participar en dicho estudio científico.
6. Acepto participar en este estudio de forma voluntaria y entiendo que puedo retirarme cuando lo crea necesario sin necesidad de motivo.

____
Firma del participante   Fecha de la firma

____
Nombre y Apellidos del participante

He informado al paciente sobre este estudio y he contestado a todas sus preguntas planteadas

____
Firma de la persona que expone el consentimiento informado   Fecha de la firma

____
Nombre y Apellidos de la persona que expone el consentimiento informado
